# A limbic circuitry involved in emotional stress-induced grooming

**DOI:** 10.1038/s41467-020-16203-x

**Published:** 2020-05-08

**Authors:** Ming-Dao Mu, Hong-Yan Geng, Kang-Lin Rong, Rong-Chao Peng, Shu-Ting Wang, Lin-Ting Geng, Zhong-Ming Qian, Wing-Ho Yung, Ya Ke

**Affiliations:** 1 School of Biomedical Sciences, Faculty of Medicine, The Chinese University of Hong Kong, Shatin, NT, Hong Kong SAR, China; 2 Laboratory of Neuropharmacology, School of Pharmacy, Fudan University, Shanghai, China; 3 Gerald Choa Neuroscience Centre, The Chinese University of Hong Kong, Shatin, NT, Hong Kong SAR, China

**Keywords:** Neural circuits, Stress and resilience

## Abstract

Prolonged exposure to negative stressors could be harmful if a subject cannot respond appropriately. Strategies evolved to respond to stress, including repetitive displacement behaviours, are important in maintaining behavioural homoeostasis. In rodents, self-grooming is a frequently observed repetitive behaviour believed to contribute to post-stress de-arousal with adaptive value. Here we identified a rat limbic di-synaptic circuit that regulates stress-induced self-grooming with positive affective valence. This circuit links hippocampal ventral subiculum to ventral lateral septum (LSv) and then lateral hypothalamus tuberal nucleus. Optogenetic activation of this circuit triggers delayed but robust excessive grooming with patterns closely resembling those evoked by emotional stress. Consistently, the neural activity of LSv reaches a peak before emotional stress-induced grooming while inhibition of this circuit significantly suppresses grooming triggered by emotional stress. Our results uncover a previously unknown limbic circuitry involved in regulating stress-induced self-grooming and pinpoint a critical role of LSv in this ethologically important behaviour.

## Abstract (web summary)

Self-grooming is a frequently observed repetitive behaviour in rodents that is believed to contribute to post-stress de-arousal. The authors identified a previously unknown limbic circuit that includes the ventral lateral septum in rats and is involved in regulating stress-induced self-grooming.

## Introduction

Stress in the form of emotional and physiological challenges is ubiquitous in everyday lives. Physical and emotional stressors can upset body homoeostasis that pertains to a steady milieu of physiological parameters as well as states of mind^1–3^. Generation of adaptive responses to stress involves the evaluation of real or perceived stressors, and the homoeostatic resolution via optimization of emotional and physiological adaptations^1,4,5^. Behavioural adaptations could include increased arousal, attention and vigilance^1^. On the other hand, maladaptive response to stress has been linked to the aetiology of anxiety, depression and a variety of other neuropsychiatric conditions^6–8^. Therefore, strategies evolved to cope with stress are essential for health and survival.

Stress in animals, including human, often results in grooming and other repetitive behaviours such as circling and rocking^9–12^. These displacement activities are believed to have adaptive values. Indeed, self-grooming is a frequently observed repetitive behaviour in rodents that serve functions more than hygiene maintenance and thermoregulation. This behaviour may represent adaptive response to stress, or restraining force that prevents over-response to stress, such as post-stress de-arousal^11–14^. Thus, unravelling the mechanism of stress-induced self-grooming is highly valuable towards understanding the neurobiological basis of stress management.

In the mammalian brain, a number of brain areas have been implicated in the generation of grooming behaviour. These include the basal ganglia^13,15^, brain stem^13,16^ and cerebellum^13,17^ that represent the downstream, mechanical motor pathways. On the other hand, some components of the limbic system, including the hypothalamus, amygdala and orbitofrontal cortex, participate in the regulation of grooming. For example, focal activation of specific hypothalamic nuclei could evoke robust grooming^18–20^, and control of self-grooming versus social behaviour by distinct amygdala neuronal subpopulations had been demonstrated^21^. Also, repeated stimulation of the orbitofrontal-striatal pathway could generate compulsive grooming^22^. Many previous studies that specifically link stress to grooming focused on the neuroendocrine system, namely the hypothalamic–pituitary–adrenocortical axis^12,13,23–25^, in accordance with the observation that central administration of some stress-related neuropeptides like corticotropin-releasing hormone (CRH), adrenocorticotrophic hormone and melanocyte-stimulating hormone could elicit grooming^26,27^, and CRH neurons orchestrate post-stress behaviours including grooming^12^. More recently, the neural pathways involved in different stress-related responses including grooming have begun to be revealed^12,14,24,25^. For example, a hypothalamic-septal pathway that mediates the influence of emotional states on grooming, escape and feeding behaviour was identified^24^. However, the relationship between stress and self-grooming is likely to be complex^13,14,28^, and the neural circuit basis of stress-induced grooming is still unresolved. In particular, the complete neural circuitry that conveys perceived stress, especially those with strong emotional component, that lead to grooming is yet to be uncovered.

In this study, by identifying and dissecting stress-related neural circuit, we revealed a previously unknown limbic circuit linking the hippocampal ventral subiculum (VS), ventral division of lateral septum (LSv) and lateral hypothalamus that regulates stress-induced self-grooming. Optogenetic activation of this di-synaptic circuitry triggered delayed but robust grooming with patterns closely resembling those evoked by emotional stress. A distinct feature of activation of this circuit is its association with a clear positive valence, unlike many other studies on grooming behaviour. In addition, we found that the neural activity of LSv precedes and is necessary for emotional stress-induced grooming while targeted functional inhibition of this circuitry suppressed grooming caused specifically by stressful paradigms. Our results thus advance our understanding of the neural circuit basis of repetitive behaviour particularly relevant to the adaptation to emotional stress.

## Results

### Activation of ventral subdivision of LS triggers robust grooming behaviour

**Fig. 1:**
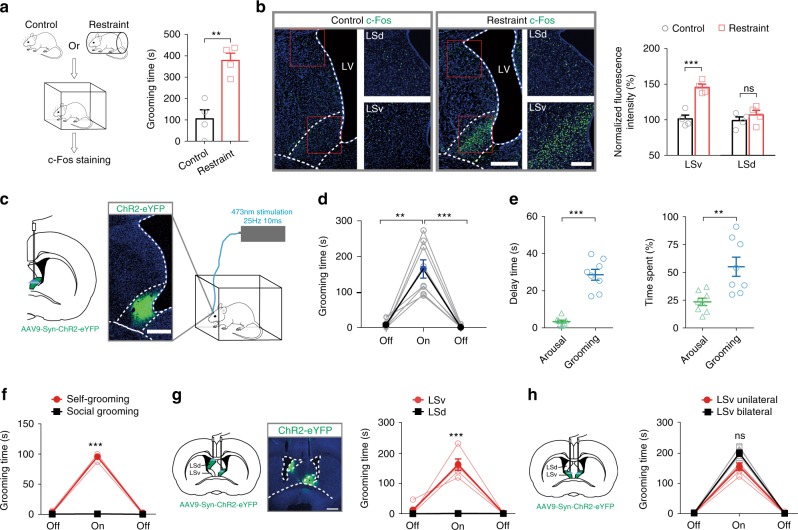
Activation of ventral subdivision of LS triggers delayed but robust grooming behaviour. **a** Body restraint imposed on rats for 20 min induced significantly increased grooming behaviour within 10 min compared with control, before sacrífice for c-Fos staining. *n* = 4; **P = 0.0024; Student’s unpaired two-tailed *t*-test. **b** Representative c-Fos staining revealing activation of neurons in LSv but not LSd following body restraint stress. Magnified LSd and LSv regions (red squares) are shown on the right. Scale bar, 500 μm (left), 200 μm (right). **c** Optogenetic activation of LSv by targeting an optic fibre at unilateral LSv that expressed ChR2-eYFP after injection with AAV9-hSyn-hChR2(H134R)-eYFP. Scale bar 500 μm. **d** A 5-min off +5-min on +5-min off optogenetic activation paradigm showed that activation of LSv neurons induced increased time spent in grooming behaviour (*n* = 8, One-way repeated measures ANOVA with Tukey post-hoc test, pre-off vs. on, **P = 0.0019; on vs. post-off, ***P = 0.0008). **e** Comparison of the delay and time spent in grooming and arousal behaviours, including rearing and heading, in the initial 2 min of LSv stimulation. Delay time, ***P < 0.0001; Time spent, **P = 0.0041; Student’s paired two-tailed *t*-test. **f** LSv optogenetic stimulation only induced self-grooming but not social grooming in the rat (comparison during light on period between the two behaviours: *n* = 3, Student’s paired two-tailed *t*-test, ***P < 0.0001). **g** Left panel: implantation of optic fibres targeting LSd and LSv in contralateral sides of the brain that expressed ChR2-eYFP. Right panel: only blue light stimulation of LSv (*n* = 4) but not LSd (*n* = 4) resulted in increased time spent in grooming. Scale bar, 1000 μm. (comparison during light on period between the two groups: Student’s paired two-tailed *t*-test, ***P < 0.0001). **h** No significant (ns) difference in the time spent in grooming between unilateral (*n* = 5) and bilateral (*n* = 4) LSv stimulation (comparison between the two groups during light on period: *P* = 0.0553, Student’s two-tailed *t*-test). All data are presented as mean ± SEM. See also Supplementary Table 1 for further statistical information. Source data are provided as a Source Data file.

We began by examining the c-Fos expression in the rat brain limbic system after imposing body restraint, a well-known stressor^29–33^, to the animals for an extended period of time, viz 20 min (*n* = 4). This protocol induced significantly increased time spent in grooming by the animals (Fig. 1a). A robust c-Fos expression was found in the LSv. Notably, the c-Fos expression was mainly confined to the ventral LSv but not in the dorsal lateral septum (LSd) (Fig. 1b, left panel). Significantly increased c-Fos signals was found in LSv compared with the control animals not receiving body restraint treatment (Fig. 1b, right panel). To probe the consequence of activating LSv neurons, after micro-injection of AAV9-Syn-ChR2-eYFP into LSv (Fig. 1c), delivery of blue light onto LSv for 5 min could trigger robust grooming (Fig. 1d; Supplementary Movie 1). At the same time, although grooming behaviour dominated during light stimulation, it exhibited a characteristic delay of several tens-of-seconds while rearing-like arousal behaviour was frequently observed prior to the occurrence of grooming (Fig. 1e). We also confirmed that self-grooming rather than social grooming was induced by LSv activation by placing a littermate in the same arena during optogenetic stimulation (*n* = 3, Fig. 1f and Supplementary Movie 2).

Consistent with the c-Fos finding, by injecting AAV9-Syn-ChR2-eYFP in LSv and LSd separately in the same animal, only optogenetic activation of LSv but not LSd induced grooming (Fig. 1g; Supplementary Movie 1), confirming the specific involvement of LSv. Furthermore, when comparing the effects of unilateral vs. bilateral stimulation of LSv, the latter induced more time spent in grooming but to a modest extent only (Fig. 1h), indicating that activities of unilateral LSv is sufficient to generate the full expression of self-grooming. Taken together, these results suggest that LSv is involved in the manifestation of repetitive self-grooming that is closely related to stress.

### Upstream and downstream nuclei of LSv contributing to self-grooming

**Fig. 2:**
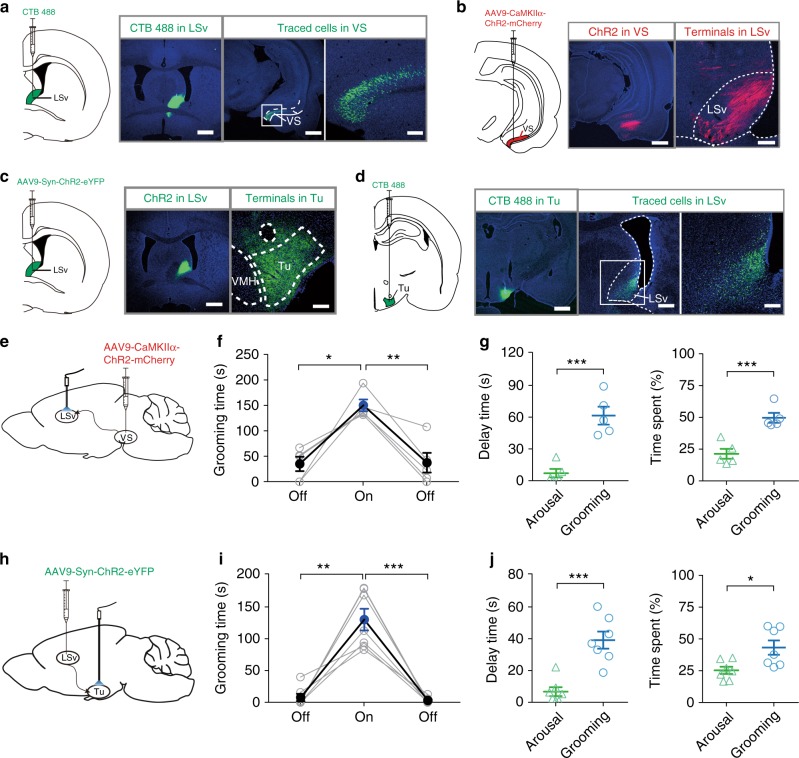
Mapping upstream and downstream nuclei of LSv contributing to self-grooming. **a** Retrograde labelling by CTB-488 microinjection in the LSv resulted in prominent expressions in the ventral subiculum (VS) of the hippocampal formation in the ipsilateral brain. Scale bar, 1000 μm. Inset shows enlarged ventral subiculum. Scale bar, 200 μm. **b** Anterograde tracing by injection of AAV9-CamIIKα-ChR2-mCherry confirmed the VS-LSv projection. Left, scale bar, 1000 μm; Right, scale bar, 200 μm. **c** Anterograde tracing by AAV9-hSyn-hChR2(H134R)-eYFP microinjection in the LSv resulted in prominent expressions in the tuberal nucleus (Tu) of the lateral hypothalamus. Left, scale bar, 1000 μm; Right, scale bar, 200 μm. **d** Injection of CTB-488 into Tu confirmed the LSv-Tu projection. Left, scale bar, 1000 μm; middle, scale bar 500 μm; Right, scale bar 200 μm. **e** and **f** Optogenetic activation of the VS → LSv pathway for 5 min at 25 Hz induced increased grooming behaviour that was significantly higher than those in the 5-min pre-light and post-light-off periods (*n* = 5, One-way repeated measures ANOVA with Tukey post-hoc test, *F* (1.489, 5.954) = 13.05, *P* = 0.0083; off vs. on, *P = 0.0189; on vs. post-off, **P = 0.0096). **g** Comparison of the delay and time spent in grooming and arousal behaviours, including rearing and heading, in the initial 2 min of stimulation. *n* = 5; delay time, ***P = 0.0004; time spent, ***P = 0.0009; Student’s paired two-tailed *t*-test. **h** and **i** Optogenetic activation of the LSv → Tu pathway for 5 min induced increased grooming behaviour that was significantly higher than those in the 5-min pre-light-off and post-light-off periods. (*n* = 7, one-way repeated measures ANOVA with Tukey post-hoc test, *F* (1.104, 6.622) = 39.73, *P* = 0.0004; pre-off vs. on, **P = 0.0025; on vs. post-off, ***P = 0.0010). **j** Comparison of the delay and time spent in grooming and arousal behaviours, including rearing and heading, in the initial 2 min of stimulation. (*n* = 7; delay time, ***P = 0.0002; time spent, **P = 0.0147; Student’s paired two-tailed *t*-test). All data are are presented as mean ± SEM. Source data are provided as a Source Data file.

To dissect the circuit underlying LSv-regulated grooming, we mapped the upstream and downstream areas of LSv. Injection of the retrograde tracer chlorea toxin b (CTB) 488 into LSv resulted in fluorescent signals in a number of brain regions, but a highly discrete and prominent labelling of the VS of the ipsilateral hippocampus was found (Fig. 2a). In agreement, anterograde labelling confirmed that VS is an upstream region of LSv (Fig. 2b). We also micro-injected AAV9-Syn-ChR2-eYFP into LSv and observed conspicuous fluorescent nerve terminals in the lateral hypothalamus tuberal nucleus (Tu, Fig. 2c). Consistently, CTB-488 injection in Tulabelled cells in LSv (Fig. 2d). Based on these observations, we performed experiments to specifically manipulate these LSv-connected upstream and downstream pathways. First, we injected AAV9-CaMKIIα-ChR2-mCherry into VS and a light cannula was implanted onto LSv (Fig. 2e). Optogenetic stimulation of the terminals of the excitatory VS → LSv pathway located in LSv at a frequency of 25 Hz triggered self-grooming behaviour similar to those of stimulating LSv (Fig. 2f), including arousal behaviour (Fig. 2g). In brain slices obtained from these animals showing grooming response to optogenetic stimulation in vivo, activation of the ChR2-expressing VS neurons generated depolarizing response leading to firing (Supplementary Fig. 1a, b). In voltage-clamp recording, the corresponding light-evoked excitatory postsynaptic currents (oEPSCs) was sensitive to 10 μM CNQX (Supplementary Fig. 1c). While tetrodotoxin (TTX, 1 μM) completely eliminated the oEPSC, addition of 4-amino-pyridine (4-AP, 1 mM) restored them (Supplementary Fig. 1d), indicating that this connection is monosynaptic in nature.

In a separate set of experiments, after injection of AAV9-Syn-ChR2-eYFP into the LSv (Fig. 2h), optogenetic stimulation of the LSv → Tu pathway by targeting the LSv terminals in Tu induced self-grooming (Fig. 2i). Again, the delay and the time spent in grooming and arousal were similar to that of stimulating LSv (Fig. 2j). In brain slice experiments, the functionality of ChR2 in LSv neurons was validated (Supplementary Fig. 1e, f). Furthermore, as LSv is populated mainly by GABAergic neurons^34^, we also confirmed in brain slice the GABAergic, monosynaptic connection from LSv to Tu in contributing to grooming (Supplementary Fig. 1g, h).

### Positive valence of LSv-associated grooming behaviour

**Fig. 3:**
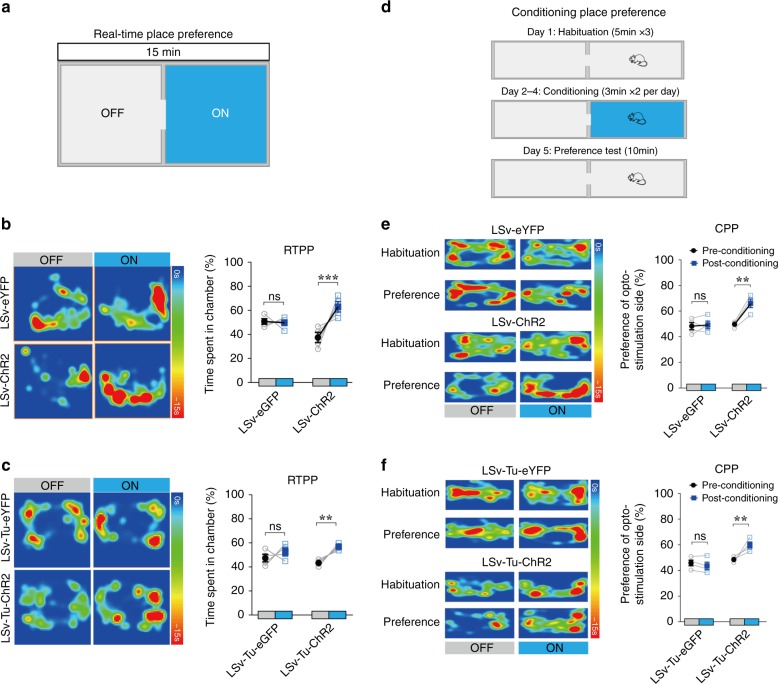
Positive valence of LSv-associated grooming behaviour. **a**–**c** Schematics and results of real-time place preference (RTPP) test based on a dual chamber setup showing increased time spent by an animal in the chamber that was associated with optogenetic stimulation of the LSv (**b**, Interaction; *F*(1, 6) = 8.908, *P* = 0.0245; Virus main effect; *F*(1, 6) = 1.589e^−031^, *P* > 0.9999; optostimulation main effect; *F*(1, 6) = 34.35, *P* = 0.0011; ***P = 0.0005) or LSv → Tu pathway (**c**, Interaction; *F*(1, 6) = 4.789, *P* = 0.0712; virus main effect; *F*(1, 6) = 2.139e^−030^, *P* > 0.9999; optostimulation main effect; *F*(1, 6) = 10.40, *P* = 0.0180; **P = 0.0043), confirmed by statistical analysis shown in the right panel in which the time spent in grooming was subtracted. Control animals received injection of AAV5-hSyn-eGFP. *n* = 4; ns: not significant; Two-way repeated measures ANOVA with Sidak post-hoc test. **d**–**f** Schematics and results of conditioning place preference (CPP) test based on a dual chamber setup. Animals spent significantly higher amount of time in the stimulation-associated chamber in the preference test for LSv (**e** interaction; *F*(1,6) = 19.86, *P* = 0.0043; virus main effect; *F*(1,6) = 7.353, *P* = 0.0350; optostimulation main effect; *F*(1, 6) = 25.24, *P* = 0.0024; **P = 0.0011) or LSv → Tu pathway (**f** interaction; *F*(1, 6) = 15.84, *P* = 0.0073; virus main effect; *F*(1, 6) = 12.81, *P* = 0.0116; optostimulation main effect; *F*(1, 6) = 7.159, *P* = 0.0368; **P = 0.0073), confirmed by statistical analysis shown in the right panel. Control animals received injection of AAV5-hSyn-eGFP. *n* = 4; ns: not significant; two-way repeated measures ANOVA with Sidak post-hoc test. All data are presented as mean ± SEM. Source data are provided as a Source Data file.

One important question is whether the grooming modulated by LSv and its output pathway signifies a positive or negative emotional state. When we assessed the intrinsic desirability vs. averseness of LSv-modulated grooming by real-time place preference (RTPP), we found significant preference for the animals to stay in the compartment associated with stimulation of the LSv (Fig. 3a, b) or LSv → Tu pathway (Fig. 3a, c), after subtracting the time spent in grooming. To reduce the influence of grooming itself in the assessment, we also conducted a conditioning place preference (CPP) test. Similarly, after 2 × 3 min of association per day between photo-stimulation and one chamber for 3 days, the animals spent significantly higher amount of time in the stimulation-associated chamber in the preference test (Fig. 3d–f). Therefore, LSv → Tu triggered grooming is clearly associated with a positive affective valence.

### The VS → LSv → Tu di-synaptic limbic circuit modulates self-grooming

**Fig. 4:**
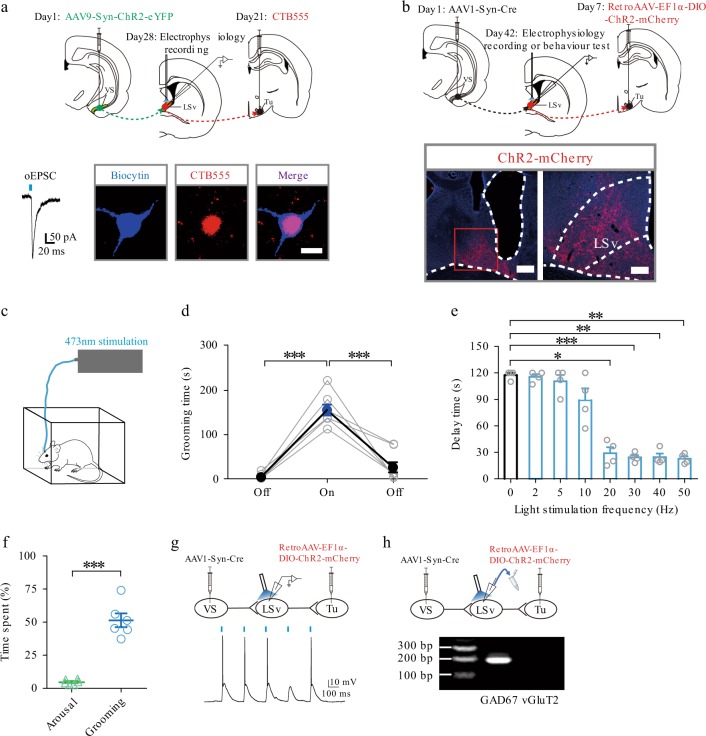
The VS → LSv → Tu disynaptic limbic circuit modulates grooming behaviour. **a** Upper panel: injection paradigm to confirm that neurons in LSv innervated by VS are neurons that send signals to Tu. AAV9-Syn-ChR2-eYFP was injected into VS and CTB-555 was injected into Tu. Lower panel: An example of a biocytin-tagged LSv neuron that generated EPSCs in response to stimulation of VS → LSv terminals was also labelled by CTB-555. Scale bar, 10 μm. **b** Experimental strategy to elucidate the role of VS → LSv → Tu circuitry in grooming. Upper panel: AAV1-Syn-Cre was injected into VS and retroAAV-EF1α-DIO-ChR2-mCherry was injected into Tu. An optical fibre was implanted onto LSv. Lower panel: as a result, only neurons in LSv that simultaneously received innervation from VS and projected to Tu could express mCherry as confirmed by confocal microscopy. Left, scale bar, 500 μm; Right, scale bar, 200 μm. **c**–**e** In these rats, in vivo light delivery onto LSv activated the VS → LSv → Tu pathway specifically in vivo **c** and significantly increased grooming behaviour (**d**, *n* = 8, *F*(1.260, 8.822) = 64.89, *P* < 0.0001, one-way repeated measures ANOVA with Tukey post-hoc test; pre-off vs. on, ***P < 0.0001; on vs. post-off, ***P = 0.0006) in a frequency-dependent manner (**e**, *n* = 4, *F*(1.517, 4.551) = 63.70, *P* = 0.0006, one-way repeated measures ANOVA with Tukey post-hoc test; 0 vs. 20 Hz, *P = 0.0134; 0 vs. 30 Hz, ***P < 0.0001; 0 vs. 40 Hz, **P = 0.0013; 0 vs. 50 Hz, **P = 0.0020). **f** Time spent in arousal and grooming behaviours in the initial 2 min of stimulation of the VS → LSv → Tu circuitry. *n* = 6; Time spent, ***P < 0.0001; Student’s paired two-tailed *t*-test. **g** Validation of functional ChR2 was confirmed by in vitro optogenetic stimulation in whole-cell recording following in vivo experiments. **h** Single-cell RT-qPCR result of cytoplasmic content of patched LSv neurons was positive to GAD67 but negative to vGluT2. All data are presented as mean ± SEM. Source data are provided as a Source Data file.

Having established the role of the VS → LSv and LSv → Tu projections in triggering self-grooming behaviour, we asked whether the neurons in LSv that receive command from the VS are the same neurons conveying grooming-related signals to the downstream Tu. To address this question, we first injected AAV9-Syn-ChR2-eYFP into VS and CTB-555 into Tu (Fig. 4a, upper panel) and prepared brain slices for whole-cell recordings targeting LSv neurons that were tagged with biocytin during recording. It was found that many neurons that generated EPSCs in response to photo-stimulation of VS terminals also expressed CTB signal (Fig. 4a, lower panel), confirming the presence of a di-synaptic VS → LSv → Tu pathway. To confirm its function, we adopted a strategy that allowed us to manipulate this specific pathway in vivo. By injecting anterograde, synapse-crossing AAV1-Syn-Cre^35^ in VS and the retrograde retroAAV-EF1α-DIO-ChR2-mCherry in Tu (Fig. 4b upper panel), only LSv neurons that were innervated by VS and also projected directly to Tu would express ChR2-mCherry (Fig. 4b, lower panel). In these animals, light delivered to LSv (Fig. 4c) induced robust self-grooming (Fig. 4d), with delays dependent on the frequency of light stimulation (Fig. 4e). Interestingly, rearing and heading behaviours were observed much less frequently (Fig. 4f; Supplementary Movie 3), suggesting that the exploratory behaviour per se is not mediated by the VS → LSv → Tu circuitry. In brain slices obtained from these animals, photo-stimulation evoked membrane depolarization of ChR2-mCherry-positive LSv neurons leading to firing (Fig. 4g). Furthermore, single-cell RT-PCR of cytoplasmic content extracted from LSv neurons was positive for GAD67 mRNA but not the glutamate neuron marker vGluT2, confirming their GABAergic nature (Fig. 4h).

### VS → LSv → Tu-induced grooming resembles those caused by emotional stress

**Fig. 5:**
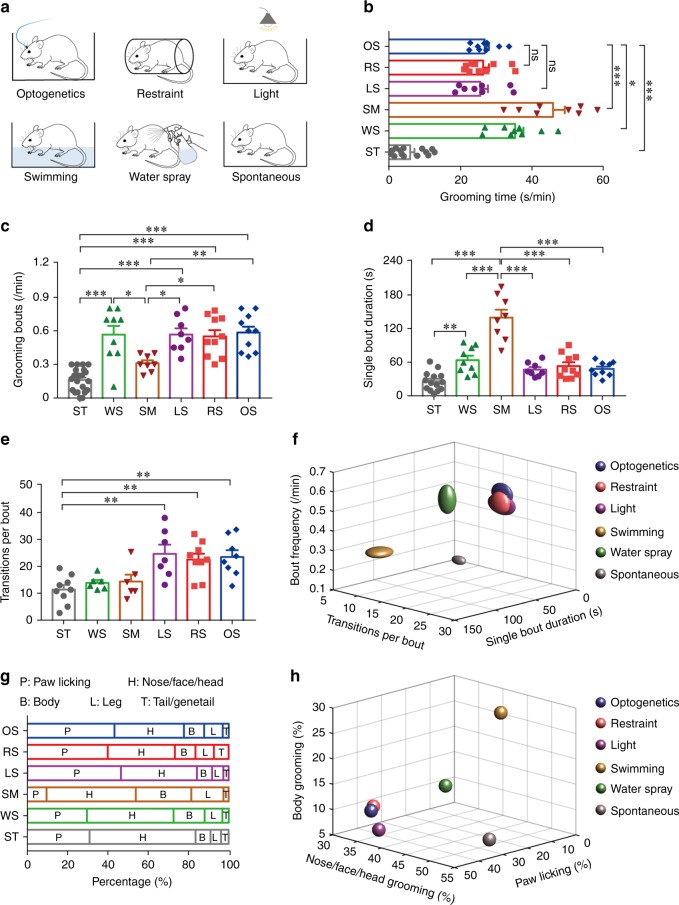
Grooming induced by optogenetic activation of the VS → LSv → Tu circuitry resembles emotional stress-induced grooming. **a** The six grooming models in this study, including optogenetic activation of LSv (OS), body restriant (RS), light exposure (LS), swimming (SM), water spray (WS) and spontaneously occurring (ST). **b** The OS-induced (*n* = 9), RS-induced (*n* = 10) and LS-induced (*n* = 8) grooming models spent similar average time in this behaviour but were significantly lower than that in SM-induced (*n* = 8) and WS-induced (*n* = 9) models. *n* = 14 for ST model (one-way ANOVA with Tukey post-hoc test; ns: not significant; OS vs. ST, ***P < 0.0001; OS vs. WS, *P = 0.0387; OS vs. SM, ***P < 0.0001). **c**–**e** The grooming frequency (bouts per min, **c**), single bout duration **d** and transitions per bout **e** were variable among the different models. One-way ANOVA with Tukey post-hoc test. *P < 0.05; **P < 0.01; ***P < 0.001. **f** Optogenetics-induced grooming, the body restraint and light exposure models are highly similar in term of bout frequency, bout duration and transitions per bout. The dimension of the symbol along an axis is defined by the SEM of the corresponding parameter. **g** The average number of times spent on grooming different body parts in the six experimental models of this study, expressed as % of total. **h** 3-D plot of the number of times spent in grooming different body parts revealed higher similarity of the optogenetics model and the restraint and light models. All data are presented as mean ± SEM. See also Supplementary Table 2 for further statistical information. Source data are provided as a Source Data file.

Next, we asked whether this VS → LSv → Tu pathway really is involved in stress-induced self-grooming. We exploited the fact that grooming behaviour are context-sensitive and could be reflected in their sequence patterns or microstructure^36,37^. To do so, we generated four additional grooming models that are related to physical and emotional challenges in different degrees, including those following free swimming^38^, water spray^25^, bright light exposure^37^ and body restraint^29,31,32^ (Fig. 5a and see “Methods” section). The former two models provoke more physical stress via moistening of the fur while the latter two models are often employed as models implicating emotional stress^30–33^.

In agreement to previous reports^13,14,36,37^ and our prediction, compared with spontaneous grooming, the four induced grooming models exhibited higher percentages of incorrect phase transition and interrupted bouts implicating elevated stress levels (Supplementary Fig. 2a, b). Interesting, for some parameters, our LSv-optogenetics stimulation model shares similarity with the light exposure and body restraint models. These include total time spent in grooming (Fig. 5b), and also the combination of bout frequency (Fig. 5c), duration of individual bouts (Fig. 5d) and the transitions per bout (Fig. 5e). Combination of these parameters together suggested a higher similarity of the optogenetics model with the body restraint and light exposure model (Fig. 5f and Supplementary Fig. 2c–e). In addition, rats in these three models also spent a higher frequency in paw licking while in the two fur moistening models they spent a higher frequency in grooming the head and body instead (Fig. 5g and Supplementary Fig. 2f–h). A plot of the number of times spent in different body parts also implicate the similarity among these models (Fig. 5h and Supplementary Fig. 2i–k).

**Fig. 6:**
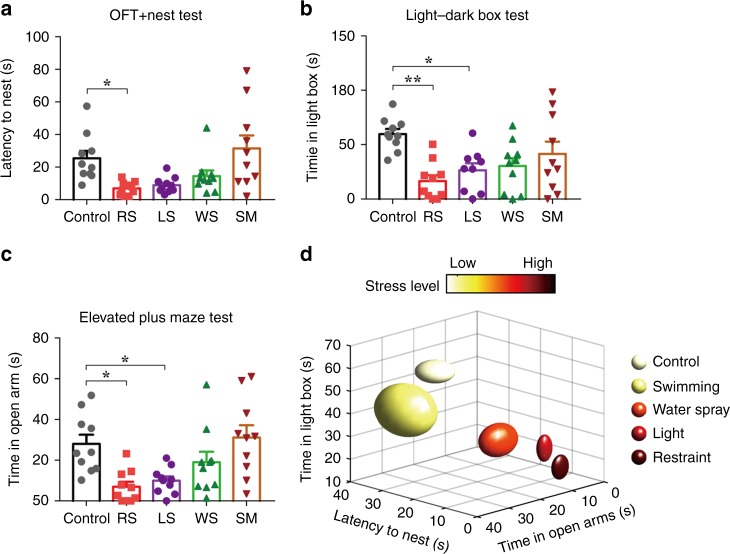
Assessment of stress levels associated with different grooming models. **a**–**c** The latency to nest in open field test (OFT) (**a**, *F*(4, 45) = 5.525, *P* = 0.0010; Control vs. RS, *P = 0.0451), the time in light box during the light–dark box test (**b**, *F*(4, 45) = 5.252, *P* = 0.0015; Control vs. RS, **P = 0.0011; Control vs. LS, *P = 0.0137) and the time in the open arms during the elevated plus maze test (**c**, *F*(4, 45) = 6.001, *P* = 0.0006; Control vs. RS, *P = 0.0113; Control vs. LS, *P = 0.0404) recorded in different models indicate significant differences mainly between the body restraint and light exposure models with the control group. *n* = 10 rats for each group; One-way ANOVA with Tukey post-hoc test. **d** 3-D plot of these parameters confirms that the body restraint paradigm was associated with the highest level of stress, which was followed by light exposure and water spray. Least stress was associated with free swimming and the control group. All data are presented as mean ± SEM. Source data are provided as a Source Data file.

To validate a higher level of stress associated with the body restraint and light exposure models, we conducted a series of tests to measure stress levels in the different models, including a new latency to nest in open field test (Fig. 6a), a light–dark box test (Fig. 6b), and the elevated plus maze (EPM) test (Fig. 6c). These were conducted after stress induction but before full expression of grooming behaviours. Combining the results of the tests (Fig. 6d) confirms that the body restraint paradigm is associated with the highest level of stress, which is followed by light exposure and water spray. Least stress is associated with free swimming and the control group. Interestingly, although water sprayed to the head or body alone rather than the whole body resulted in more time spent in grooming the head or body as expected (Supplementary Fig. 3a), these two models cluster well with the whole body water spray model in terms of bout frequency/transitions per bout/single bout duration analysis (Supplementary Fig. 3b).

### Microstructures of grooming triggered by stressors and the VS → LSv → Tu circuit

**Fig. 7:**
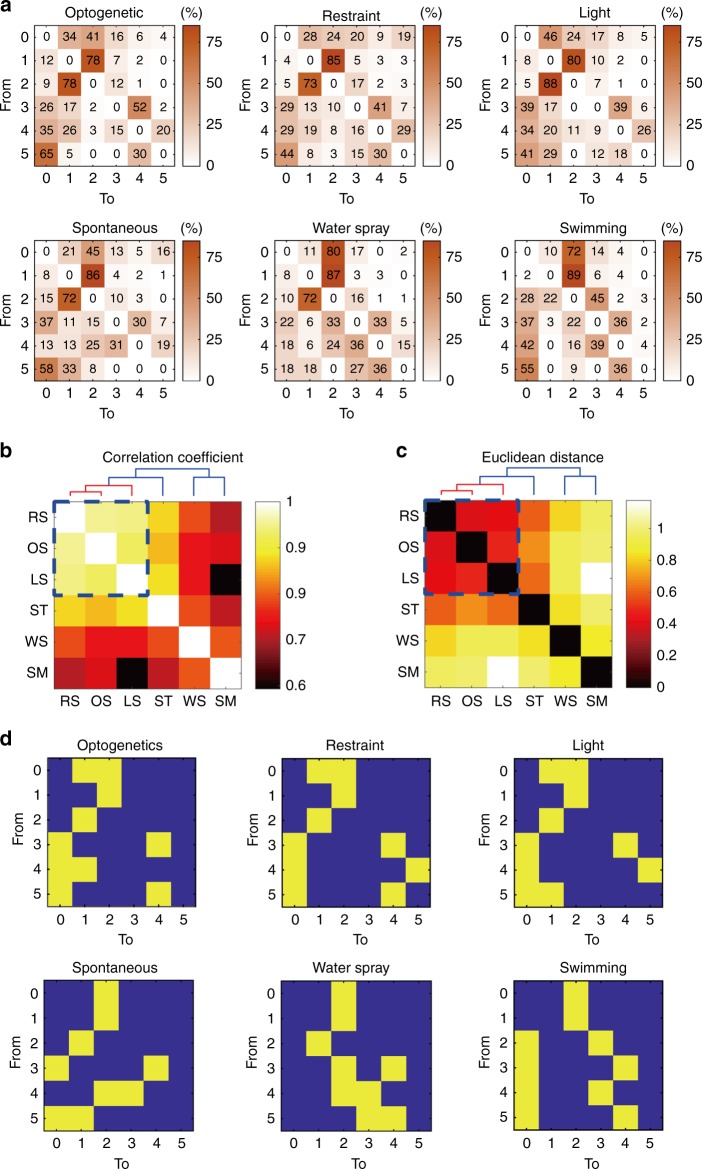
Microstructure analysis of grooming under different contexts. **a** The transition probability matrices summarizing the likelihood from one grooming phase to another phase in the six grooming models. The different phases of no grooming/grooming activities were defined as no grooming (phase 0), paw licking (phase 1), nose/face/head grooming (phase 2), body grooming (phase 3), leg grooming (phase 4) and tail/genital grooming (phase 5). The numbers show the occurrence in % and are colour-coded. **b**, **c** The correlation coefficients **b**, and Euclidean distances **c** between each pair of transition probability matrices were derived. Hierarchical clustering analysis supports a higher similarity of the optogenetics model and the restraint and light models. RS body restraint stress, OS optogenetics stimulation of LSv, LS bright light exposure, ST spontaneous grooming, WS water spray, SM swimming. **d** The patterns and microstructures of six grooming models summarized by the phase transition probability matrix shown in **a** were transformed into two-colour binary format using a threshold of 25% illustrating higher similarity among the optogenetics model, the restraint model and the light model.

It has been suggested that the patterns, or microstructure, of grooming is variable and may reflect differences in the context and underlying neural mechanisms^13,36,37^. But despite increased emphasis on the significance of analyzing grooming structures^13,36^, sensitive methods that can distinguish different context-dependent grooming are still not available. Here, we developed an alternative approach to address this question. Instead of focusing on arbitrarily defined ‘incorrect’ transition of different grooming phases, we analysed the frequencies of all possible transitions among different phases (Supplementary Fig. 4a) and then calculated the percentage of each transition and expressed as a phase transition probability matrix (Fig. 7a). It is obvious that the phase transition matrices derived from different models are by no means uniform. To quantitatively determine the similarity among the matrices, two quantitative indices, namely, the cross-correlations and the Euclidean distances were derived. The pairwise comparisons indicate that the optogenetics model and the light exposure and body restraint models have the highest cross-correlations (Fig. 7b) and the least Euclidean distances (Fig. 7c). Hierarchical clustering analyses also confirmed their higher resemblance. In fact, when these matrices were transposed into binary-coded format (Fig. 7d) or presented as phase connection graphs highlighting the most probable transitions and prominent phase (Supplementary Fig. 4b) the similarities among the optogenetics, light exposure and restraint models are obvious.

### Increased activity of LSv neurons precedes emotional stress-induced grooming

**Fig. 8:**
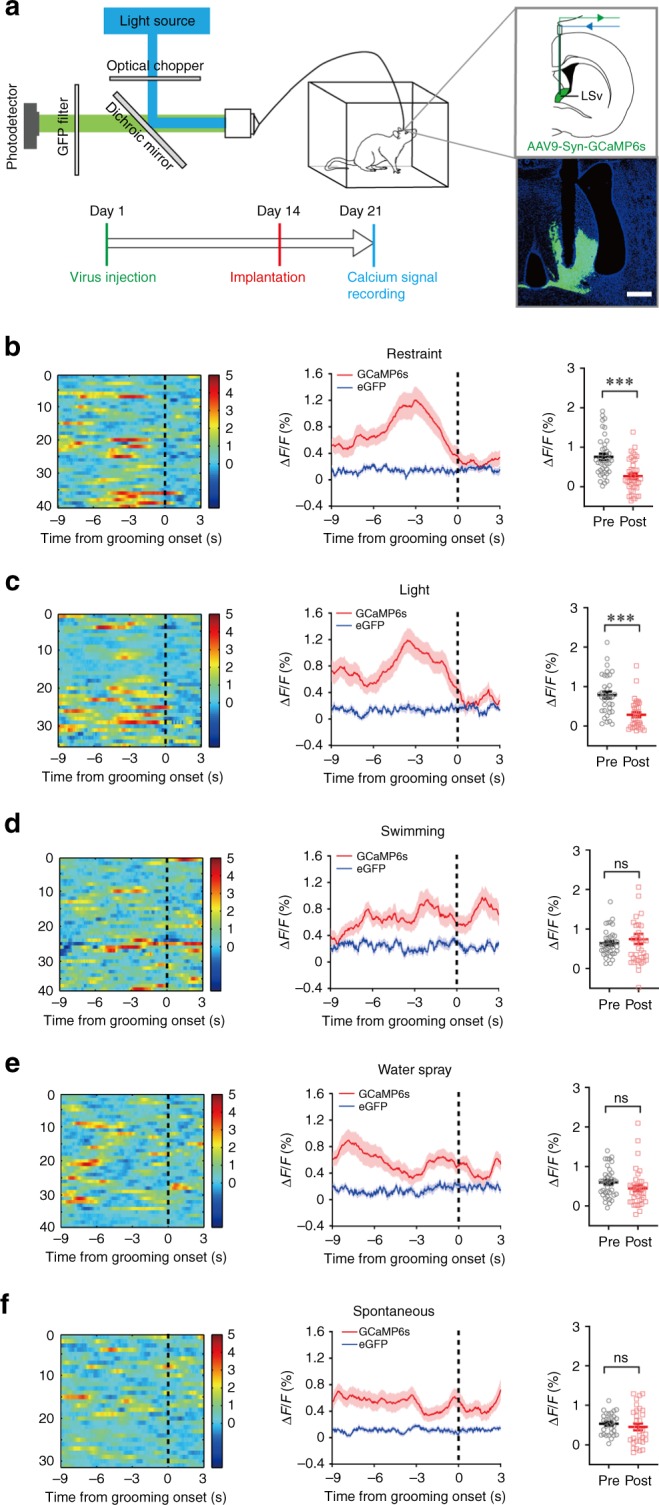
Activation of LSv neurons precedes emotional stress-induced grooming. **a** Setup of fibre photometry to record calcium activity from LSv neurons infected with AAV9-Syn-GCaMP6s or AAV5-hSyn-eGFP virus. The correct expression of GCaMP6s and placement of the fibre optics were verified post-mortem. Scale bar 500 μm. **b**–**f** Typical results of calcium activity in LSv around the start of grooming in multiple bouts (left panel) and the averaged results (red traces, middle panel) in the body restraint model (**b**; *n* = 40 trails from five rats; ***P < 0.0001), light exposure model (**c**; *n* = 40 trails from four rats; ***P < 0.0001), swimming model (**d**; *n* = 40 trails from four rats), water spray model (**e**; *n* = 40 trails from six rats) and spontaneous grooming (**f**; *n* = 40 trails from four rats). There was significant rise in calcium activity (Δ*F*/*F*) prior to the start of the grooming in the body restraint and light exposure models but not the other models as shown in the right panels. ns non-significant; ns: not significant; Student’s paired two-tailed *t*-test. The blue traces represent signals from animals which expressed only eGFP but not GCaMP. All data are presented as mean ± SEM. Source data are provided as a Source Data file.

To gain further insight into the role of LSv in stress-mediated self-grooming behaviour, we determined the population dynamics of LSv neurons in freely behaving rats in the different grooming models by fibre photometry based on the GCaMP6s reporter (Fig. 8a). After stress induction, the calcium activities of the neurons were monitored and aligned to the start of detected grooming bouts. In the body restraint (Fig. 8b) and light exposure model (Fig. 8c), we detected on average a clear rise in calcium signals of LSv neurons shortly before the start of a grooming event. Significant differences in Δ*F*/*F* in the pre-grooming and post-grooming periods were found (Fig. 8b, c). In contrast, no discernible changes in calcium signals were found in the swimming (Fig. 8d) and water spray (Fig. 8e) models as well as in spontaneous grooming (Fig. 8f). In addition, the fluorescent signals in control animals expressing eGFP in the LSv neurons showed no change during the grooming behaviour.

### Inhibition of the VS → LSv → Tu circuit suppressed grooming caused by emotional stress

**Fig. 9:**
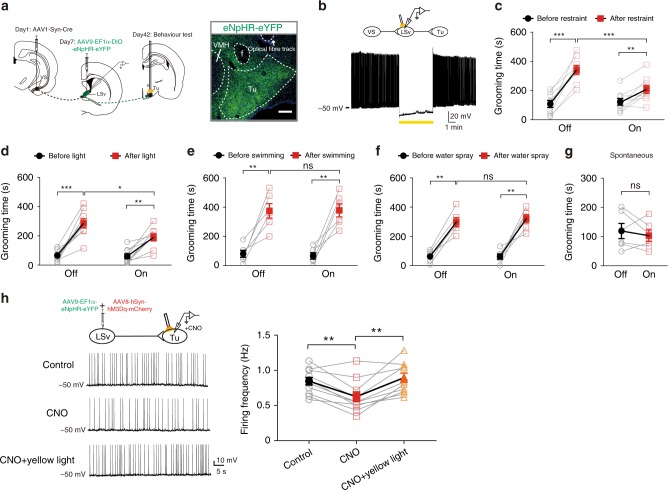
Activation of LSv neurons is necessary for emotional stress-induced grooming. **a** Schematics for optogenetics manipulation. Targeted functional inhibition of the VS → LSv → Tu circuitry was achieved by injection of AAV1-Syn-Cre into VS, AAV9-EF1a-DIO-eNpHR-eYFP into LSv and implantation of optical fibre onto Tu. Scale bar 500 μm. **b** Whole-cell recordings from LSv neurons in brain slices obtained from these animals validated eNpHR-mediated inhibition via prolonged (5 min) yellow light delivery. **c**–**f** While significant increases in grooming time were found in light-off control group following body restraint (**c**, *n* = 9) and light exposure (**d**, *n* = 9), the increases in grooming time were significantly smaller when light was delivered. In contrast, the increases in grooming time after swimming (**e**, *n* = 6) and water spray (**f**, *n* = 6) were similar in both light-off and light-on trials. ns not significant; *P < 0.05; **P < 0.01; ***P < 0.001, two-way repeated measures ANOVA with Sidak post-hoc test. **g** Optogenetic inhibition did not affect the time that the animal spent in spontaneous grooming. *n* = 6, *P* = 0.4861; Student’s paired two-tailed *t*-test. **h** Targeted functional activation and inhibition LSv terminals in Tu by injection of AAV9-EF1a-eNpHR-eYFP and AAV8-hSyn-hM3Dq-mCherry into LSv and recording in Tu neurons. The spontaneous firing of Tu neurons induced by −50 mV holding level can be partially inhibited by applying CNO and recovered by yellow light stimulation. (*n* = 11, one-way repeated measures ANOVA with Tukey post-hoc test; **P < 0.01). All data are presented as mean ± SEM. See also Supplementary Table 3 for further statistical information. Source data are provided as a Source Data file.

Finally, to establish the causal relationship between activation of VS → LSv → Tu and self-grooming, we applied optogenetic to achieve targeted inhibition of this circuitry. We injected AAV1-Syn-Cre into VS and AAV9-EF1α-DIO-eNpHR-eYFP into LSv, which allowed the VS → LSv → Tu pathway to be inhibited specifically by targeting the NpHR-expressing terminals in Tu (Fig. 9a). The expression and function of eNpHR in LSv neurons were verified in brain slices (Fig. 9b). When yellow light was delivered into Tu, there was still increased self-grooming observed in body restraint and light exposure models but the level was suppressed (Fig. 9c, d). Nevertheless, the level of grooming was significantly weaker than when the light was off, confirming a major contribution of this pathway in post-stress grooming. In contrast, light delivery did not affect the time spent in grooming in both the swimming and water spray models (Fig. 9e, f). Spontaneous grooming was also unaffected (Fig. 9g). We performed control study in which non-functional AAV5-hSyn-eGFP was injected into VS, LSv, and Tu. No abnormality was found in these animals, including grooming activities, when light was delivered (Supplementary Fig. 5). To validate the effect of optogenetic inhibition of LSv → Tu terminals on activity of Tu neurons, AAV-expressing eNpHR were injected into LSv as before. At the same time, we also injected AAV8-hSyn-hM3Dq-mCherry into LSv (Fig. 9h). In subsequent brain slice experiments, we patched Tu neurons and held them at a membrane potential whereas spontaneous firing occurred. Under this condition, CNO superfusion applied to activate LSv neurons suppressed the firing rate of Tu neurons, which was accompanied by membrane hyperpolarization, in agreement with increased GABA release from LSv terminals (Fig. 9h). Under this condition, when yellow light is shone on the LSv terminals aiming to inhibit GABA release, the decreased firing of Tu neurons was rectified (Fig. 9h). Parallel experiments were performed based on chemogenetic inhibition of LSv neurons in the VS → LSv → Tu pathway (Supplementary Fig. 6a), with results supporting the same conclusion (Supplementary Fig. 6b–f).

## Discussion

Grooming in mammals represents an important adaptive response to stress, and provides a valuable model for elucidating the brain mechanism of stress management. In this study, we have identified a di-synaptic hippocampal–septal–hypothalamus circuit in the limbic system of rodent brain that regulates self-grooming but not social-grooming behaviour. Through a combination of tract tracing, optogenetic and electrophysiological experiments, we showed that in this circuitry, LSv neurons that receive monosynaptic excitatory innervation from VS in turn send monosynaptic GABAergic projection to Tu neurons in the hypothalamus. There were different studies in the past that implicate the involvement of the subiculum, the LSv and various hypothalamus regions in processing stress or mediating stress-induced responses^39–43^. A recent study integrating gene expression and brain-wide connectivity in the mouse also revealed a hippocampal-septo-hypothalamic network pertinent to cognitive-limbic integration^44^. However, our study reveals the precise connections among the specific sub-divisions of these three nuclei that plays a critical role in modulating stress-induced response. Also, the grooming behaviour we observed distinguishes from those of many previous studies in its strong association with emotional stress, positive rather than negative valence and a delayed instead of immediate response following stimulation.

Among the different stress-induced grooming models studied, we showed by fibre photometry that only the body restraint and light exposure protocols activate LSv neurons with a peak activity preceding grooming, and that optogenetic and chemogenetic inhibition of the discrete VS → LSv → Tu circuit suppressed grooming triggered specifically by these two paradigms. A body of evidence suggests that these two paradigms evoke mainly emotional stress^30–33^ rather than the physical stress as in the swimming and water spray models^30,45^. In agreement, our own assessment confirmed that the body restraint and light exposure paradigms are associated with a higher level of stress when compared with the water spray and swimming models. Our findings therefore strongly implicate that the di-synaptic circuit from VS to Tu regulates grooming particularly relevant to emotional stress. It is noted that optogenetic and chemogenetic inhibition of this pathway could not completely suppress the post-stress grooming. It is likely that additional pathway exists to effect similar response.

It has been suggested that analysis of grooming activity and its microstructure may serve as a useful index of stress and anxiety^13,36,37^. In this study, we found that activation of the VS → LSv → Tu circuit triggered excessive grooming with microstructures closely resembling those evoked by the restraint and light exposure paradigms. We analysed the probabilities of all possible transitions among different stages of grooming, and revealed subtle but discernible differences in the microstructures of grooming driven by different types of stressors. This finding affirms that the grooming microstructure is highly related to the specific context that triggers the behaviour^13,14,28,37^. It is noteworthy that, according to our analysis result, the microstructure of spontaneous grooming is more similar to restraint-induced and light exposure-induced grooming rather than the two water-associated models. This finding may reflect that the physical stress of body wetting generated relatively unique patterns of grooming different from other types of grooming. At the same time, although spontaneous grooming occurs at a much lower frequency, this behaviour may encompass those that are generated by internal drive and motivational elements related to psychological need akin to emotional stress-induced grooming. On the whole, our approach represents a valuable addition to the currently available methods in analyzing context-dependent microstructure of grooming. However, caution should be taken in interpreting the results as the microstructure could also be affected by other factors like the method that induces grooming.

If the circuitry we identified is involved in the manifestation of emotional stress-induced response, it seems counter-intuitive that the grooming response is associated with a positive valence, as demonstrated by our RTPP and CPP results. Indeed, Xu et al. ^24^ had demonstrated negative emotional state associated grooming response following activation of a hypothalamic-septal pathway. Behavioural response to stress often manifests as heightened arousal, attention and alertness supported by concomitant physiological adaptations, and believed to be associated with a negative emotional state. However, these responses are often time-limited due to restraining forces that prevent over-response that could be harmful^4,12^. The desirability of the LSv → Tu pathway-modulated post-stress grooming behaviour implicates a calming effect, which is consistent with an adaptive displacement activity to stress, or suppression of over-response to stress. In fact, this pathway may not be responsible for generating stress-induced emotional state itself, but only for initiating grooming action that helps to maintain a sense of well-being.

As the limbic component of the hippocampus, the subiculum has been suggested to exert a generalized up-stream influence on integration of limbic functions^46,47^ probably via its innervation to various limbic forebrain structures^44,48^. Indeed, the ventral division of subiculum, VS, may play a role in stressor selection with respect to regulation of neuroendocrine response to stress^39,40,49,50^. However, whether VS exerts an excitatory or inhibitory influence on the hypothalamo-pituitary-adrenocortical axis is not clear^39,40^. In relation to this, our findings not only extend the understanding of the role of VS in processing emotional stress-related information but that via its downstream LSv → Tu pathway, behavioural adaptation like grooming could be effected. On the other hand, the hypothalamus is well-known to be a primary output node for the limbic system^49,51^, mediating many innate behaviours. Although the Tu has not been studied in great detail before, consistent with our finding, the lateral hypothalamic area including the Tu has recently been implicated in grooming behaviour, including the development of pathological grooming^12,25,52^. Since a number of other hypothalamic areas are known to be involved in grooming behaviour^19,20,53^, the relationship of Tu with respect to these other areas remains to be investigated.

The LSv is a limbic structure long known to be associated with a variety of cognitive and emotional processes^41,51^. For example, it has been implicated for the modulation of anxiety^54^, expression of fear-conditioning to context^55^, and regulation of autonomic responses to aversive stimuli^32^. The impact of the LSv on stress-related behaviour is however controversial^56–60^, partly due to that previous studies relied on studying the effects of lesion or electrical stimulation and therefore were non-specific. Many of these studies also considered the LSv as a single nucleus, despite evidence for the presence of heterogenous neuronal populations constituting different sub-regions^61,62^. Our results revealed that only the ventral subdivision of the LSv, and more specifically its GABAergic neuronal population, conveys emotional stress relevant to the generation of repetitive grooming behaviour. Based on our findings, we propose that LSv receives emotional stress-related information from VS and in turn regulates down-stream Tu in triggering grooming.

After stimulation, the typical tens-of-seconds of delay before grooming takes place is puzzling. This is in sharp contrast to immediate grooming behaviours triggered by stimulating some other brain areas^12,24,25,52,53^, e.g. the dorsomedial hypothalamus and the orbitofrontal-striatal projection^22^ that are reminiscent of compulsive-like behaviour. One interpretation is that a build-up time is needed for the manifestation of the response, which is consistent with an integration or decision-making role of LSv. At the same time, we found that stimulation of LSv neurons and related pathways could trigger other behaviours, notably rearing-like arousal behaviour. In contrast, when the VS → LSv →Tu pathway is activated specifically, the grooming is almost free of preceding arousal behaviour. This dissociation between arousal and grooming strongly suggests that while LSv is involved in arousal/exploratory behaviour, this is mediated by microcircuitries other than the VS → LS →Tu pathway that controls grooming per se. Thus, we speculate that as an integration hub, LSv is composed of heterogenous neuronal subpopulations that might map different inputs to different innate behaviours. Indeed, Xu et al.^24^ found that LSv also receives emotional state-related signals from the PVN, and triggers responses including grooming, escape behaviour and suppression of feeding, with negative rather than positive valence. Our findings therefore enrich the central role of LSv in the fine regulation and coordination of different innate behaviors. As the output pathway of LSv to Tu is GABAergic, the nature of the interaction between these inhibitory neurons with neurons in Tu should be clarified in future studies.

As aberrant response to stress is regarded as a factor driving compulsive repetitive behaviour in some neuropsychiatric disorders^6,7,10^, notably autism^63,64^ and obsessive-compulsive disorders^22,65,66^, our findings therefore not only uncover a limbic circuit that plays a significant role in emotional stress response but also provide a basis for deciphering the complete circuit of emotional processing and their malfunctions that could lead to abnormal repetitive behaviours in different brain disorders.

## Methods

### Animals

Adult male Sprague-Dawley (SD) rats weighing 300–320 g were used in this study. The animals were bred and maintained by the Laboratory Animal Service Centre of The Chinese University of Hong Kong (CUHK). The animal room was controlled at a temperature of 23 °C on a 12-h light/dark cycle. All animals were handled in strict accordance with the CUHK guidelines and the procedures were approved by the Animal Experimentations and Ethics Committee. All experiments were performed during the light phase (09:00–19:00).

### Viral constructs

Adeno-associated viruses (AAV) including AAV5-hSyn-eGFP, AAV9-hSyn-hChR2(H134R)-eYFP, AAV9-CaMKIIα-hChR2(E123A)-mCherry, AAV8-hSyn-hM3Dq-mCherry, RetroAAV-EF1α-double floxed-hChR2(H134R)-mCherry-WPRE-HGHpA, AAV9-Ef1α-DIO-eNpHR3.0-eYFP, RetroAAV-hSyn-DIO-hM4Di-mCherry, AAV9-Syn-GCaMP6s-WPRE-SV40 were all purchased from Addgene (Watertown, USA). All viral titre were >5 × 10^12^ particles per ml.

### Stereotaxic surgeries for in vivo studies

SD rats were anesthetized with ketamine (75 mg/kg, i.p.) and xylazine (6 mg/kg, i.p.), and placed gently in a stereotaxic frame (Narashige, Tokyo). For micro-injections, a Hamilton syringe (33-gauge) filled with AAV virus or tracer was unilaterally or bilaterally placed into the target area according to the corresponding coordinates: LSv (−0.12 mm A/P, ±1.0 mm M/L, 6.0 mm D/V), VS (−5.40 mm A/P, ±4.0 mm M/L, 9.2 mm D/V), and Tu (−3.24 mm A/P, ±1.4 mm M/L, 9.2 mm D/V) from dura. 0.1–0.5 μl virus or tracer were injected at speed of 10–50 nl/min. The needle was left in place for an additional 10 min before retraction. The scalp incision was sutured, and postinjection analgesics were given for 3 days to aid recovery. The animals were allowed at least 3 weeks to recover and express a virus before optical fibre implantation or behavioural test. For fibre photometry and optogenetics experiments, an optical fibre (200-μm core, NA = 0.48 for photometry, NA = 0.37 for optogenetics) or fluid-injection cannula (26-gauge guide cannula possessing a 32-gauge dummy cannula) was implanted directly above. The fibre or guide cannula, together with two stainless steel screws was secured to the skull using dental cement. The rats were allowed at least 1 week to recover before behavioural test. Correct location of implanted fibre/cannula was confirmed postmortem.

### Optogenetic and chemogenetic manipulations

For ChR2 photostimulation, 473-nm light laser (10 ms, 25 Hz, unless otherwise indicated, Newdoon Technology) was delivered via an optic cable (200-μm core, 0.37 NA, Doric Lenses) and the stimulation duration varied in different experiments, as described in the relevant results. Laser power was 5 mW measured at the tip of the fibre, which was implanted 0.3 mm above the targeted nucleus. For eNpHR photoinhibition, two optical fibres were attached to the double cannula for constant illumination of the targeted site (589 nm, 10 mW from the tip of 200 μm fibre) of the targeted site. A 589-nm laser (10 mW, Newdoon Technology) was continuously turned on throughout the post-stress behaviour sessions. For chemogenetic manipulation, clozapine N-oxide (CNO) (5 μM/0.5 μl, Sigma), or vehicle (saline) was administered via the implanted cannula 40 mins before grooming was assessed. For all behavioural experiments, the animals were videotaped, and the behaviours were evaluated offline.

### Retrograde and anterograde tracing

For retrograde tracing, recombinant cholera toxin-b conjugated to AlexaFluor-488 (CTB-488, ThermoFisher) in PBS was injected to the target site at LSv. At least 7 days were allowed for complete retrograde transport before sacrifice. For anterograde tracing, AAV9-Syn-ChR2-eYFP was injected into LSv and allowed at least 4 weeks for full expression. Brain sections (30 μm) were prepared and examined under confocal laser scanning microscope (C1, Nikon). To establish the disynaptic circuit of VS → LSv → Tu, AAV9-Syn-ChR2-eYFP was injected into VS for at least 4 weeks for expression. One week prior to sacrifice, recombinant cholera toxin-b conjugated to AlexaFluor-555 (CTB-555, ThermoFisher) in PBS was injected into Tu. Coronal slices of 300 μm were prepared by using a vibrotome (Campden 5100MZ-PLUS Vibrotome) for whole-cell patch recordings. Neurons in LSv that responded to blue light stimulation with EPSC (oSPSC) were filled with biocytin. Biocytin was revealed with Alexa Fluor^TM^ 405-conjugated streptavidin–biotin complex (ThermoFisher). The expressions of biocytin and CTB-555 were examined under confocal laser scanning microscope (C1, Nikon).

### Behavioural studies

For all behavioural tests, animals were habituated for 30 min/day consecutively for 3 days before testing. The behaviours of the animals were recorded in a test chamber (30 cm length, 30 cm width and 60 cm height) by a video camera (Logitech, C922). Each animal was tested at least three times on different days to verify reproducibility.

For spontaneous grooming model, the animal was placed in the test chamber, and the spontaneous activities were recorded for 20 min.

For restraint stress-induced grooming^29,31,32^, the animal was restrained in a black tube (5 cm diameter, 25 cm length) for 20 min and then put immediately into the test chamber for 20 min of video recording.

For bright light-induced grooming, animals were transported to the dimly lit laboratory and left undisturbed for 30 min prior to exposure to the light box on the day of the experiment^37^. The animal was exposed to the bright light (60–80 W) from a lamp, 10 cm from above the home cage, for 20 min. Immediately afterward, the animal was placed in the test chamber, and its behaviour was recorded for 20 min.

For optogenetically induced grooming, the behaviour was monitored for a total of 15 min, following a 5-5-5 min protocol in which blue light pulses (473 nm, 5 mW, 10 ms each at 25 Hz) was turned on in the middle 5 min. Grooming and other behaviours were video-taped. To test for social grooming behaviour, two animals were placed in the same chamber, and the same 5-5-5 min protocol was applied.

For water spray-induced grooming^25^, the animal was sprayed with water squirts directed to the face, belly, and back (four squirts per area) with a spray bottle pre-filled with sterile water (25 °C) as the “whole-body” water spray model. For “head-only” model, water was directly sprayed to the left and right face (four squirts per side). For “body-only” model, water was sprayed to the belly and back (eight squirts per area). It was then placed in the test chamber for video recording of the behaviour for 10 min. Before testing, the animals were habituated once per day for 3 consecutive days.

For swimming-induced grooming^30,38^, the animal was placed in an open swimming pool (100 cm × 50 cm and 50 cm high) that was filled with water at 25 °C. Water depth was set at 15 cm to ensure that the rat could stand freely and allowed them to freely swim for 5 min. After at least 3 days of adaptation, animals were allowed to swim for 2 min on the day of grooming testing, and excess water was then removed and allowed to drain away before the animals were placed immediately into the test chamber for 10 min of video recording.

Based on the natural aversion of rodents towards brightly lit areas and at the same time a tendency towards exploratory behaviour^67^, the following behaviour tests were performed to evaluate the emotional status associated with different grooming-inducing paradigms.

For open-field with a nest test, a dark shelter nest (20 cm × 20 cm and 40 cm high) was put in the corner of an open field (100 cm × 100 cm and 40 cm high). Animals were handled and habituated for 10-min to the open-field 2 days before the test. On the testing day, each rat was allowed to first freely explore the box for 5 min. Then, immediately after going though the induction protocol of a grooming model, the rat was placed in the box at the corner diagonal to the nest. The latency of escape to the nest was recorded as an index of stress-like behaviour. A shorter latency indicates a higher stress level.

For light–dark box test^67^, the test box consists of two boxes (light and dark, each was 50 cm × 40 cm and 40 cm high) and connected by an open door. When testing, the animal was first placed in light box and the total time spent in light box was recorded for a period of 5 min. The less time spent in the light box indicates a higher stress level.

For EPM test, it was used to measure anxiety-related behaviour in rodents^67^. The maze was consisted of a central square (15 cm × 15 cm), two open arms without walls (15 cm × 40 cm) and two closed arms with walls (15 cm × 40 cm and 40 cm high). Each rat was placed in the centre of the maze, after which the rat behaviour was recorded for a period of 5 min. The time spent in the open arms was used as an index of stress-like behaviour.

Behavioural analysis: The grooming and other behaviours were quantified manually by three observers with the aid of a video editing software (iJianJi, Guangzhou Quying Technology). The observers were blind to the experimental conditions. Social grooming was assessed when a rat licked and chewed the fur of the conspecific, while placing its forepaws on the back or the neck of the other rat. Arousal behaviours including rearing and heading were also examined. For gross analysis of self-grooming, the number of grooming bouts, the duration of individual bout and therefore the total grooming time in the test period, were evaluated. Self-grooming was defined as when the animal licked, or used the forelimb to stroke, its own body parts including the paws, nose, eyes, head, body, legs, tail and genital. An interruption of 6 s or more separated two individual bouts^36,37^.

### Grooming patterns and microstructures

To analyse the patterns and microstructures of grooming behaviour under different conditions, different phases, or stages, of grooming activities were defined, including paw licking (phase 1), nose/face/head grooming (phase 2), body grooming (phase 3), leg grooming (phase 4), tail and genital grooming (phase 5), according to conventional protocol^36,37^. In addition, no grooming was defined as phase 0. To facilitate the identification of these patterns by the observers who were blind to the treatment, the recorded videos were replayed at 1/4 of the actual speed. By noting the time points of the start and end of these different phases, the exact sequences of the different phases within a grooming bout was determined. A grooming bout was considered interrupted if at least one pause in action (<6 s) was recorded within its transitions of phases. In addition, the transitions between different phases of grooming pattern were used to evaluate the ‘correct’ transitions based on the conventional cephalocaudal progression stereotypy: (0 → 1), (1 → 2), (2 → 3), (3 → 4), (4 → 5), and (5 → 0) and the otherwise ‘incorrect’ transitions represented by other patterns. From these data, the total number of transitions and the percentages of incorrect transitions were calculated.

To analyse the microstructures, all grooming bouts under each of the six experimental conditions were collected and pooled. Then, the transition probabilities from one phase to another phase were calculated, and a transition probability matrix was obtained. As an example, in spontaneous grooming, the animals together spent a total of 112 times in phase 1 (paw-licking). The transitions from paw-licking to other phases took place at the following frequencies: 9 (1 → 0), 96 (1 → 2), 4 (1 → 3), 2 (1 → 4) and 1 (1 → 5). The transition probabilities were therefore 8.04%, 85.71%, 3.57%, 1.79% and 0.89%, respectively. The overall patterns of phase transitions could be visually captured by the transposed transition matrix and connectivity graphs shown in Fig. 7d and Supplementary Fig. 4b.

To be able to compare quantitatively the similarity among the transition probabilities matrices, we computed two indices, namely the Euclidean distance (*D*) and Pearson’s correlation coefficient (CC) for each pair of matrices, whereas

1 $$D = \sqrt {\mathop {\sum}\nolimits_{i = 1}^n {(y_i - x_i)^2} }$$

and

2 $${\mathrm{{CC}}} = \frac{{\mathop {\sum }\nolimits_{i = 1}^n (x_i - x)(y_i - \overline y )}}{{\sqrt {\mathop {\sum }\nolimits_{i = 1}^n (x_i - \overline x )^2} \sqrt {\mathop {\sum }\nolimits_{i = 1}^n (y_i - \overline y )^2} }}$$

in which {*x*_*i*_} and {*y*_*i*_} is the corresponding elements in the two transition probability matrices and *n* is the number of elements in the matrix. Thus, *D* computes the overall disparity of the two matrices derived from the distance between the corresponding elements, so a smaller *D* indicates a higher similarity. On the other hand, a higher CC indicates that the pair of transition matrices bear a higher similarity. Then the dendrograms of hierarchical clustering by *D* and by CC were obtained using the MATLAB Statistics Toolbox functions linkage () and dendrogram (). The height of each inverted-U-shape in the dendrogram is proportional to the dissimilarity between the two nodes being connected. More closely related nodes were connected at lower levels.

### Real-time place preference

The animal was put in a box (120 × 50 cm, 40 cm high), which was divided into two equal chambers without any contextual cues^68^. One chamber was paired with a 25 Hz photostimulation and the other identical chamber was without photostimulation. The behaviour of the animal was monitored and subsequently analysed by the ANY-Maze tracking software (Version 4.7, Stoelting CO). The percentages of time that the animal spent on the stimulated and unstimulated sides of the chamber in a period of 15 min were quantified.

### Conditioned place preference (CPP)

The animals were habituated to handling for 3 days prior to the beginning of the procedure. Experiments were conducted in two interconnected chambers (60 × 40 and 40 cm high each) that could be separated by a sliding door. The two chambers were decorated with different forms of stripes on the wall. Animal movements in each chamber were recorded and analysed with the Anymaze software^69^. The procedure consisted of three stages: preconditioning (baseline), conditioning, and testing phases. On the first day, the sliding door was retracted, and rats could explore the entire apparatus freely for 15 min (3 × 5 min). Animals that spent >70% of time in either of the compartments were excluded from further analysis. Immediately following the preconditioning phase, the rats underwent conditioning in both sides, respectively. During conditioning, one of the two chambers was paired with a photo-stimulation (25 Hz of 10 ms laser pulses, 473 nm) for 2 × 3-min/day for 3 days. During the test phase, the animals did not receive any treatment and had free access to both compartments for a total time of 10 min. Animal movements in each of the chambers were recorded, and the time spent in each chamber was analysed by the Anymaze software.

### Brain slice electrophysiology and optogenetics

Adult rat brain slices were prepared as following procedures^70,71^. The rats having completed in vivo optogenetic stimulation experiments were deeply anesthetized by isoflurane, and then perfused transcardially with 100 ml room temperature NMDG artificial cerebrospinal fluid (aCSF) containing (in mM): 92 NaCl, 2.5 KCl, 1.25 NaH_2_PO_4_, 20 NaHCO_3_, 10 HEPES, 25 Glucose, 5 Na-ascorbate, 2 thiourea, 3 Na-pyruvate, 10 MgSO_4_, 0.5 CaCl_2_, 12 N-acetyl-l-cysteine, which was saturated with carbogen (95% O_2_/5% CO_2_) prior to use. After perfusion the rats were decapitated and the brains were gently extracted from the skull and placed into the cutting solution (NMDG aCSF) for an additional 30 s. Coronal sections at 300 μm were cut with a vibrotome (Campden 5100MZ-PLUS Vibrotome) and transferred into a holding chamber containing 34 °C NMDG aCSF to recover for 10 min. After the initial recovery, the slices were transferred into a new holding chamber containing HEPES holding aCSF containing (in mM): 92 NaCl, 20 NaHCO_3_, 25 Glucose, 2.5 KCl, 1.25 NaH_2_PO_4_, 10 HEPES, 5 Na-ascorbate, 2 thiourea, 3 Na-pyruvate, 2 CaCl_2_, 2 MgSO_4_, 12 N-acetyl-l-cysteine. After another hour of recover, the slices could then be transferred to the recording chamber with normal aCSF containing (in mM): 125 NaCl, 2.5 KCl, 11 Glucose, 26 NaHCO_3_, 1.25 NaH_2_PO_4_, 2 CaCl_2_, and 2 MgCl_2_.

For recording of optically evoked action potential, excitatory postsynaptic currents (oEPSCs) and spontaneous firing, we used internal solution containing (in mM): 130 mM K-gluconate, 10 mM KCl, 10 mM HEPES, 1 EGTA, 2 mM MgCl_2_, 2 mM Na2-ATP, 0.4 mM Na3-GTP. For optically evoked inhibitory postsynaptic currents (oIPSCs), the internal solution was (in mM): 50 mM KCl, 10 mM HEPES, 1 EGTA, 2 mM MgCl_2_, 2 mM Na2-ATP, 2 mM Tris GTP. Light pulses at 470 nm were delivered through a patterned light stimulator (Polygon400 DSI-E-0470-0590-NK1 Dynamic Spatial Illuminator) to activate ChR2 while recorded neurons were voltage-clamped at −70 mV. Access resistance (<20 MΩ) was not compensated and monitored continually throughout each experiment. Recordings were terminated whenever the input resistance increased >30% or access resistance exceeded 20 MΩ. Signals were acquired using a MultiClamp 700B amplifier controlled by Clampex 10.4 software via a Digidata 1550 interface (Molecular Devices). Responses were filtered at 3 kHz, digitized at 10 kHz, and analyzed using Clampfit 10.7 (Molecular Devices). In a subset of experiments, biocytin was included in the internal solution. After recording, slices were fixed overnight in 4% paraformaldehyde. Filled neurons were identified by staining using streptavidin-conjugated Alex 405 (ThermoFisher).

### Fibre photometry

To monitor the neuronal activity in LSv, the rats were injected with 100 nl of AAV9-Syn-GCaMP6s-WPRE-SV40 or AAV5-hSyn-eGFP into the right LSv. Two weeks after the virus injection, an optical fibre (NA 0.48, Newdoon Technology) was implanted to target the right LSv. With at least 1-week recovery, the optical fibre was connected to FibreOptoMeter (Plexon’s Multi-Fibre Photometry) through an optical fibre patch cord (200-μm, 0.53 NA, Doric) for photometry recording. To record fluorescence signals, a beam from a 470 LED was reflected with a dichroic mirror, focused with a lens coupled to a PMT. The LED power at the tip of the patch cord was <50 μW. Fluorescent signals were collected and synchronized with behaviour of the animal via the CineLyzer system (Plexon, Dallas, USA).

For analysis of photometry data, smoothed Δ*F*/*F* values obtained from CineLyzer system were exported to Matlab for further analysis. The data were represented by heatmaps or averaged response (shaded area indicate SEM) in a time window between 9 s before and 3 s after grooming onset. Smoothed Δ*F*/*F* values were calculated through three different steps: To begin with, the raw fluorescence was averaged to get *F*_AVG_ (*n*) over a sliding time window (0.75 s) in a symmetrical pattern. The baseline for a particular frame (*F*_BASELINE_ (*n*)) was the minimum of the *F*_AVG_ (*n*) in a time window (3 s) preceding this frame. Next, Δ*F* was calculated by subtracting *F*_BASELINE_ (*n*) from *F*_RAW_ (*n*) and then dividing by *F*_BASELINE_ (*n*). Finally, Δ*F* was smoothed with an exponentially weighted moving average. The exponential weighting was described by a time constant $\tau_0$ and the averaging window was set to 5*$\tau_0$. The frame rate of system is 30.

3 $${\mathrm{\Delta }}F/{F}({n})_{{\mathrm{unsmoothed}}} = \frac{{{F}_{{\mathrm{RAW}}}({n}) - {F}_{{\mathrm{BASELINE}}}({n})}}{{{F}_{{\mathrm{BASELINE}}}\left( {n} \right)}}$$

4 $${N}_{\tau 0} = {\mathrm{floor}}(5 \ast \tau _0 \ast {\mathrm{frame}}\,{\mathrm{rate}})$$

5 $$\Delta {F}/{F}({n})_{{\mathrm{smoothed}}} = \frac{{\mathop {\sum }\nolimits_0^{{N}_{\tau 0}} {\mathrm{\Delta }}F/{F}({n} - x)_{{\mathrm{unsmoothed}}}{\mathrm{{e}}}^{\frac{{ - \left| x \right|}}{{{N}_{\tau 0}}}}}}{{\mathop {\sum }\nolimits_0^{{N}_{\tau 0}} {\mathrm{{e}}}^{\frac{{ - \left| x \right|}}{{{N}_{\tau 0}}}}}}$$

### Histology

Rats were anaesthetized and perfused with PBS followed by 4% PFA in PBS. The brain was fixed in 4% PFA at 4 °C overnight. Fixed samples were sectioned into 30-μm coronal sections using a cryostat (ThermoFisher). For c-Fos staining, the brain samples were prepared 60 min after behavioural test followed by fixation. The brain sections were blocked in 10% normal goat serum in PBS with 0.3% Triton X-100 in PBS for 2 h and then incubated with anti-c-Fos antibody (1:2000; Cell Signaling Technology) at 4 °C overnight. After thorough rinsing in PBS, the slices were incubated with goat anti-rabbit lgG secondary antibody (1:1000, Invitrogen) in block solution. The sections were rinsed in PBS and counterstained with DAPI and mounted. Images were taken under a confocal laser scanning microscope (C1, Nikon).

### Single cell RT-qPCR

Patch pipettes were pulled and filled with 3.0–5.0 μl of nominally RNAse-free internal solution using a microloader. The tip diameter of the pipette was around 1/3 of the size of cell body. Under visual inspection, the cell was harvested into the patch pipette by applying a negative pressure. The cell mixture was put into a tube on wet ice for at least 1 min. The tube was frozen in liquid nitrogen and stored at −80 °C until the RT reaction was carried out. Synthesis of first-strand cDNA from 3 μl (for qPCR) of total RNA (in 10 μl) was carried out with the Prime Script RT reagent Kit (DRR037A, TaKaRa). qPCR was run on iCycler (Bio-Rad) by using SYBR Green Supermix (Bio-Rad). A multiplex two-round single-cell qPCR was carried out for simultaneous detection of vesicular glutamate transporters (vGluT2), glutamic acid decarboxylase 67 (GAD67). The first PCR conditions included an initial denaturation for 5 min at 94 °C, 30 cycles with 45 s denaturation at 94 °C, 45 s annealing at 61 °C and 70 s extension at 72 °C, final elongation 7 min at 72 °C. An anliquot (5 μl) of first round PCR product used as a template for the second round PCR (40 cycles) using nested primers. The conditions were the same as described for the first round. Products were identified with gel electrophoresis with GelRed nucleic acid gel stain (Biotium). The oligonucleotide primers used for single-cell RT-PCR are listed in Supplementary Table 4.

### Reproducibility

Experiments were repeated independently with similar results at least three times. Micrographic images presented in figures are representative ones from experiments repeated independently: Fig. 1b (four times), Fig. 1c (eight times), Fig. 1g (four times), Fig. 2a (three times), Fig. 2b (five times), Fig. 2c (four times), Fig. 2d (three times), Fig. 3 (four times), Fig. 4a (three times), Fig. 4b (five times), Fig. 4h (nine times), Fig. 8a (six times) and Fig. 9a (five times).

### Statistics

Statistical analysis was performed using GraphPad Prism 7.02, using unpaired two-tailed *t*-test, paired two-tailed *t*-test, one-way ANOVA with Tukey post-hoc test, two-way ANOVA unless otherwise indicated. Values are reported as mean ± standard error of the mean (SEM). The cutoff value of significance was *P* = 0.05. Electrophysiology data was analyzed using Clampfit 10.7 (Molecular Devices). All figures were prepared with Photoshop CC 2017, Adobe Illustrator CS6 (version 16), Microsoft Excel 365.

### Reporting summary

Further information on research design is available in the Nature Research Reporting Summary linked to this article.

## Supplementary information

Supplementary Information

Description of Additional Supplementary Files

Supplementary Movie 1

Supplementary Movie 2

Supplementary Movie 3

Reporting Summary

## Data Availability

All data are contained in the main text, extended data or the supplementary materials, and are available from the corresponding author upon reasonable request. The source data underlying Figs. 1a, b, 1d–h, 2f–g, 2i–j, 3b, c, 3e, f, 4d–f, 5b–e, 6a–c, 8b–f and 3c–h and Supplementary Figs. 1c, d, 1g–h, 2a, b, 2f–h, 5a–c and 6a–f are provided as a Source Data file.

## Source data

Source Data
