# Supplementary material for: A limbic circuitry involved in emotional stress-induced grooming
Source: Nat Commun. 2020 May 8;11:2261. doi: 10.1038/s41467-020-16203-x (PMC7210270; doi:10.1038/s41467-020-16203-x)
Supplement: Supplementary file 1 — Supplementary Information [file 41467_2020_16203_MOESM1_ESM.pdf]

Supplementary information for

A limbic circuitry involved in emotional stress-induced grooming

Mu et al

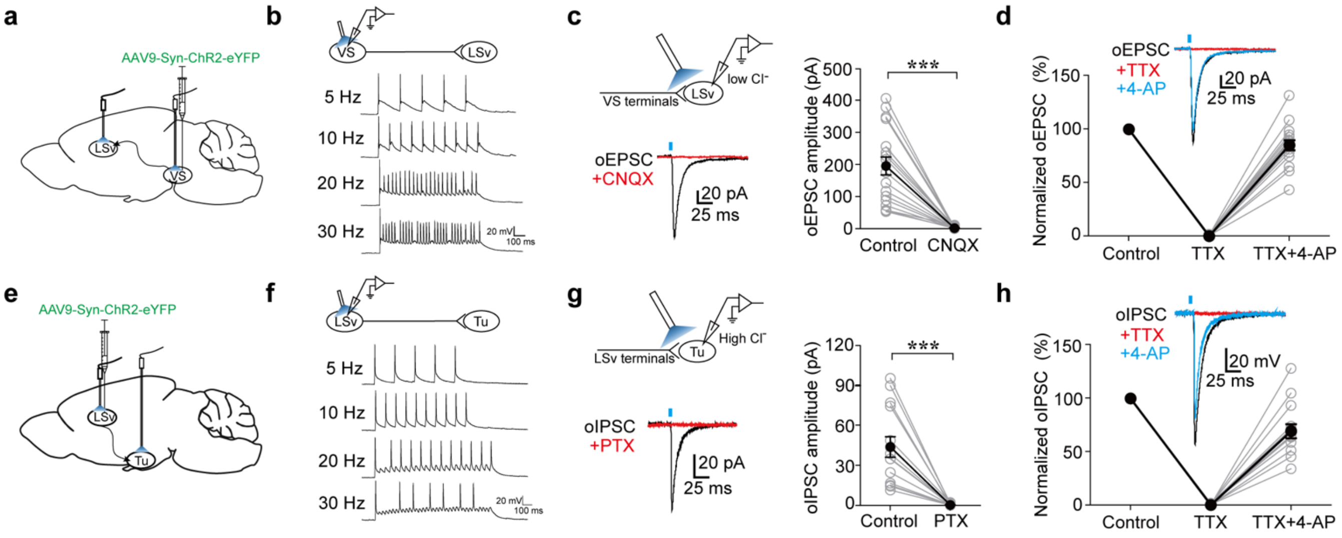

Supplementary Figure 1 | Monosynaptic glutamatergic projection from VS to LSv and GABAeric projection from LSv to Tu contributing to grooming behaviour.

a, Schematics for virus injection and optogenetic stimulation. Microinjection of AAV9-Syn-ChR2-eYFP into VS and recording at VS and LSv respectively. **b,** Patch-clamp recordings from VS neurons in brain slices responded to optical stimulation at different frequencies. **c,** The optically-induced EPSC (oEPSC) was blocked by CNQX (10 µM). n = 18 neurons from 6 rats; ****P* < 0.0001; Student's paired two-tailed t-test. **d,** TTX (1µM) completely eliminated the oEPSC while addition of 4-amino-pyridine (4-AP, 1mM) restored them. n = 17 neurons from 6 rats. **e,** Microinjection of AAV9-Syn-ChR2-eYFP into LSv and recording at LSv and Tu respectively. **f,** Patch-clamp recordings from LSv neurons in brain slices responded to optical stimulation at different frequencies leading to firing. **g,** A Tu neuron was held at a holding potential more negative than E_{Cl} .The optically-induced IPSC (oIPSC) was blocked by picrotoxin (PTX, 100 nM). n = 14 neurons from 4 rats, ****P* < 0.0001; Student's paired two-tailed t-test. **h,** TTX (1 µM) completely eliminated the oIPSC while addition of 4-amino-pyridine (4-AP, 1mM) restored them. n = 14 neurons from 4 rats. All data are presented as mean ± SEM. Source data are provided as a 'Source Data File'.

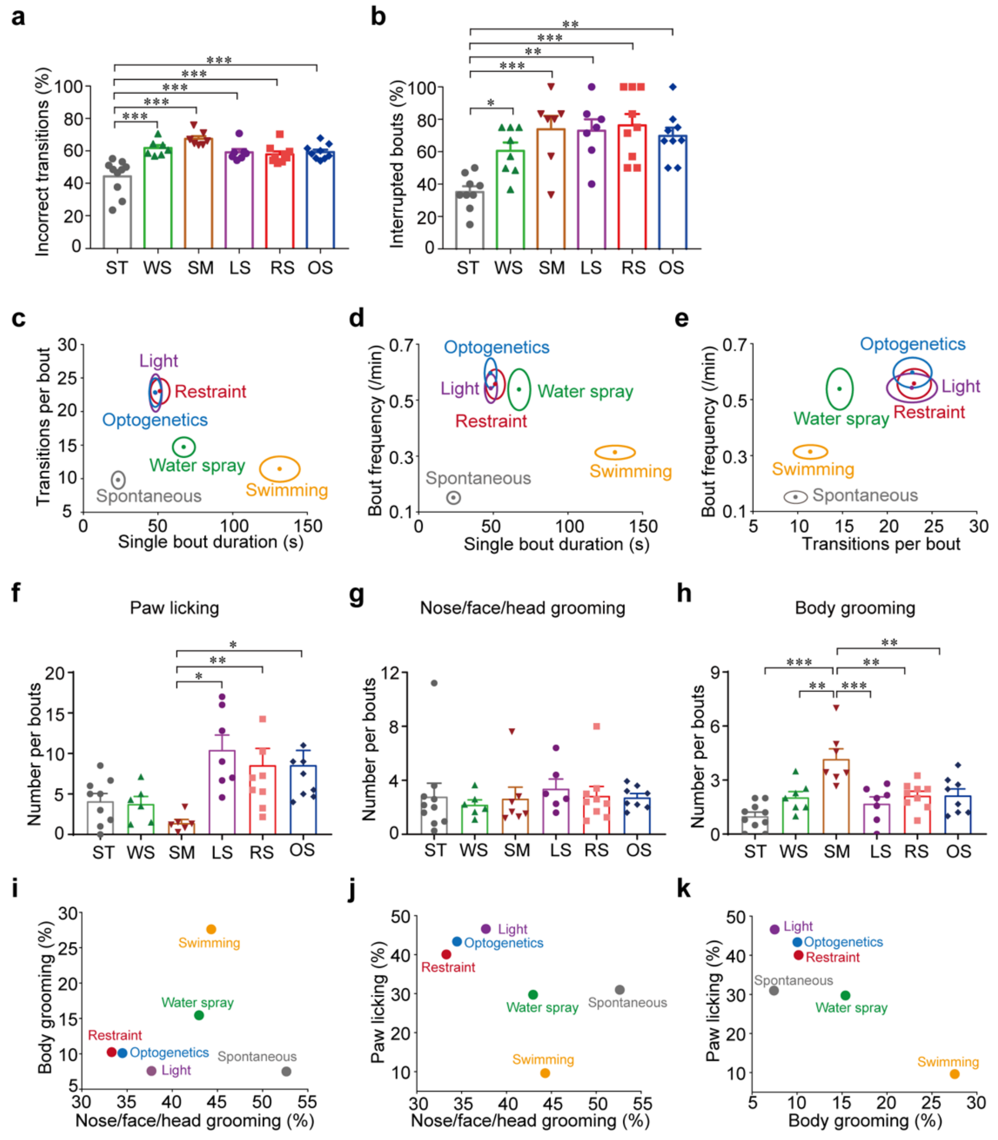

Supplementary Figure 2 | Gross grooming structures suggesting similarity among the LSv optogenetics, body restraint and light exposure grooming models.

a, Quantification of incorrect transitions with respect to the cephalocaudal sequence of stereotypic grooming patterns in the different grooming models (n = 10 for spontaneous group; n = 7 for water spray group; n = 7 for swimming group; n = 7 for light exposure group; n = 9 for body restraint group; n = 9 for optogenetic group; One-way ANOVA with Tukey *post-hoc* test; $F(5, 43) = 11.85$, $P < 0.0001$; ST vs. RS, ***$P = 0.0008$; ST vs. LS, ***$P = 0.0006$; ST vs. WS, ***$P < 0.0001$; ST vs. SM, ***$P < 0.0001$; ST vs. OS, ***$P = 0.0002$). **b,** Number of interrupted bouts, defined as a pause of grooming action for less than 6 seconds, in the different grooming models (n = 10 for spontaneous group; n = 7 for water spray group; n = 7 for swimming group; n = 7 for light exposure group; n = 9 for body restraint group; n = 9 for optogenetic group; One-way ANOVA with Tukey *post-hoc* test; $F(5, 43) = 7.072$, $P < 0.0001$;

ST vs. RS, ***P = 0.0001; ST vs. LS, **P = 0.0011; ST vs. WS, *P = 0.0443; ST vs. SM, ***P = 0.0008; ST vs. OS, **P = 0.0014). **c-e,** Corresponding 2D plots of Fig 5f showing the resemblance of the optogenetics model with the body restraint and light exposure models when examined by the grooming frequency, single bout duration and transitions per bout. **f-h,** Quantification of the number of bouts spent on paw licking **(f,** $F(5, 40) = 4.47$, $P = 0.0025$; SM vs. RS, *P = 0.0420; SM vs. LS, **P = 0.0086; SM vs. OS, *P = 0.0410**),** nose/face/head grooming **(g,** $F(5, 40) = 0.2016$, $P = 0.9599$**)**, and body grooming **(h,** $F(5, 42) = 9.251$, $P < 0.0001$; ST vs. SM, ***P < 0.0001; RS vs. SM, **P = 0.0019; LS vs. SM, ***P = 0.0003; WS vs. SM, **P = 0.0024; SM vs. OS, **P = 0.0029**)** in the 6 different grooming models. ns: not significant; n = 9 rats for spontaneous group, n = 6 rats for water spray group, n = 6 rats for swimming group, n = 7 rats for light exposure group, n = 9 rats for body restraint group, n = 9 rats for optogenetic group; One-way ANOVA with Tukey *post-hoc* test. **i-k,** Corresponding 2D plots of Fig. 5h showing the resemblance of the optogenetics model with the body restraint and light exposure models when examined by these parameters. All data are presented as mean ± SEM. Source data are provided as a ‘Source Data File’.

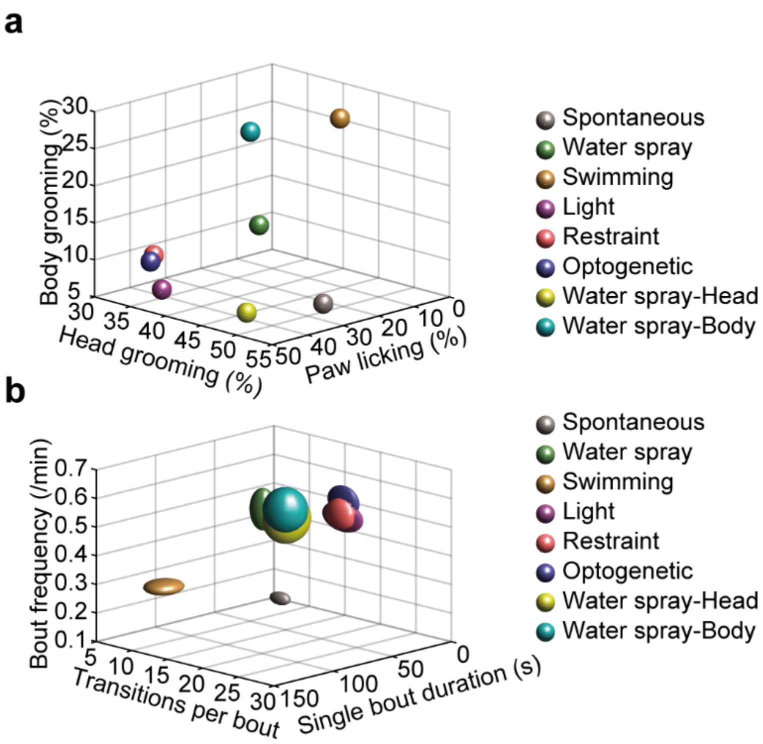

Supplementary Figure 3 | Structure of grooming behavior induced by water spray to the head or body alone cluster with the whole body water spray model.

a, 3-D plot showed water spraying to the head or body alone rather than the whole body resulted in more time spent in grooming the head or body as expected. **b,** Grooming behavior induced by water spray to the head or body alone cluster well with the whole body water spray model in terms of bout frequency/transitions per bout/single bout duration analysis. The dimension of the symbol along an axis is defined by the SEM of the corresponding parameter. Source data are provided as a 'Source Data File'.

a

Optogenetics

from \ to	0	1	2	3	4	5	Total
0	0	36	43	17	6	4	106
1	35	0	226	20	7	1	289
2	24	217	0	33	4	0	278
3	21	14	2	0	42	2	81
4	23	17	2	10	0	13	65
5	13	1	0	0	6	0	20
Total	116	285	273	80	65	20	

Restraint

from \ to	0	1	2	3	4	5	Total
0	0	25	21	18	8	17	89
1	10	0	203	13	7	6	239
2	12	177	0	42	5	7	243
3	27	12	9	0	39	7	94
4	22	15	6	12	0	22	77
5	27	5	2	9	18	0	61
Total	98	234	241	94	77	59	

Light

from \ to	0	1	2	3	4	5	Total
0	0	29	15	11	5	3	63
1	20	0	191	24	4	1	240
2	10	186	0	14	2	0	212
3	21	9	0	0	21	3	54
4	12	7	4	3	0	9	35
5	7	5	0	2	3	0	17
Total	70	236	210	54	35	16	

Spontaneous

from \ to	0	1	2	3	4	5	Total
0	0	8	17	5	2	6	38
1	9	0	96	4	2	1	112
2	19	92	0	13	4	0	128
3	10	3	4	0	8	2	27
4	2	2	4	5	0	3	16
5	7	4	1	0	0	0	12
Total	47	109	122	27	16	12	

Water spray

from \ to	0	1	2	3	4	5	Total
0	0	6	44	4	0	1	55
1	15	0	164	5	5	0	189
2	25	174	0	39	3	2	243
3	14	4	21	0	21	3	63
4	6	2	8	12	0	5	33
5	2	2	0	3	4	0	11
Total	62	188	237	63	33	11	

Swimming

from \ to	0	1	2	3	4	5	Total
0	0	12	83	16	5	0	116
1	1	0	47	3	2	0	53
2	47	38	0	77	3	5	170
3	44	4	26	0	43	2	119
4	24	0	9	22	0	2	57
5	6	0	1	0	4	0	11
Total	122	54	166	118	57	9	

b

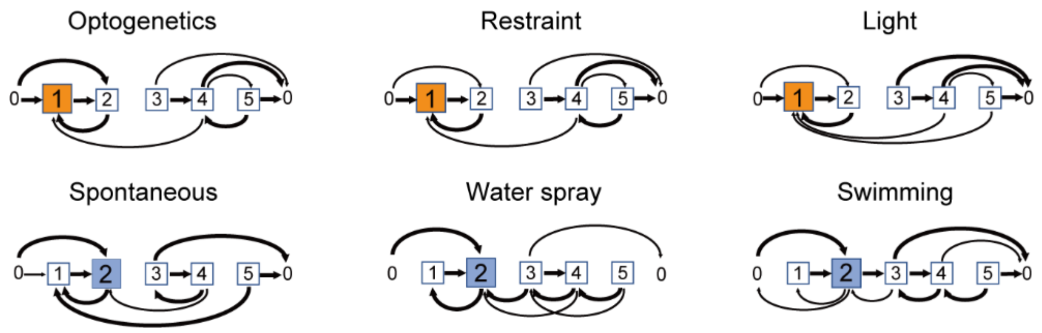

Supplementary Figure 4 | Microstructure analysis of different grooming models.

a, The number of all possible phase transitions recorded in different grooming models. The different phases of no grooming/grooming activities were defined as no grooming (phase 0), paw licking (phase 1), nose/face/head grooming (phase 2), body grooming (phase 3), leg grooming (phase 4) and tail/genital grooming (phase 5). **b,** Similarity among different grooming models visualized by the phase connection graphs highlighting the major transitions among the different phases. The main phase that the rats spent in each model is also highlighted. Thin lines: transitions >20%; thick lines: transitions >30%.

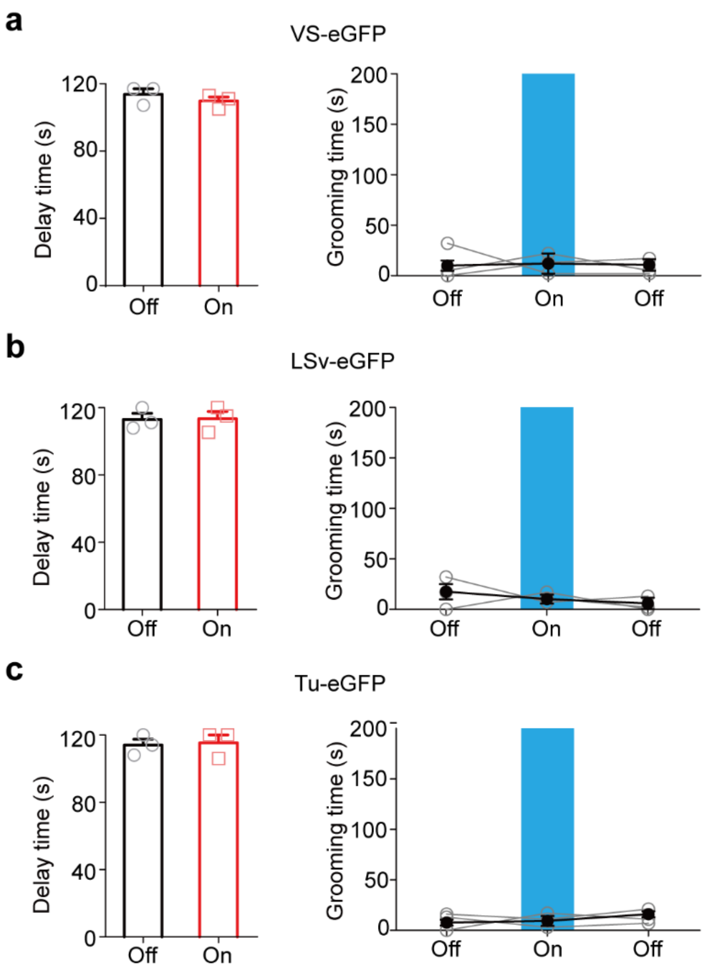

Supplementary Figure 5 | Lack of effect of control viruses on grooming behaviour. Control animals were injected with non-functional AAV5-hSyn-eGFP into **a,** (n = 3) ventral subiculucm (VS), **b,** (n = 3) ventral division of lateral septum (LSv) and **c,** (n = 3) tuberal nucleus (Tu). No abnormality was found in these animals, including grooming activities, when light was delivered. All data are presented as mean ± SEM. Source data are provided as a 'Source Data File'.

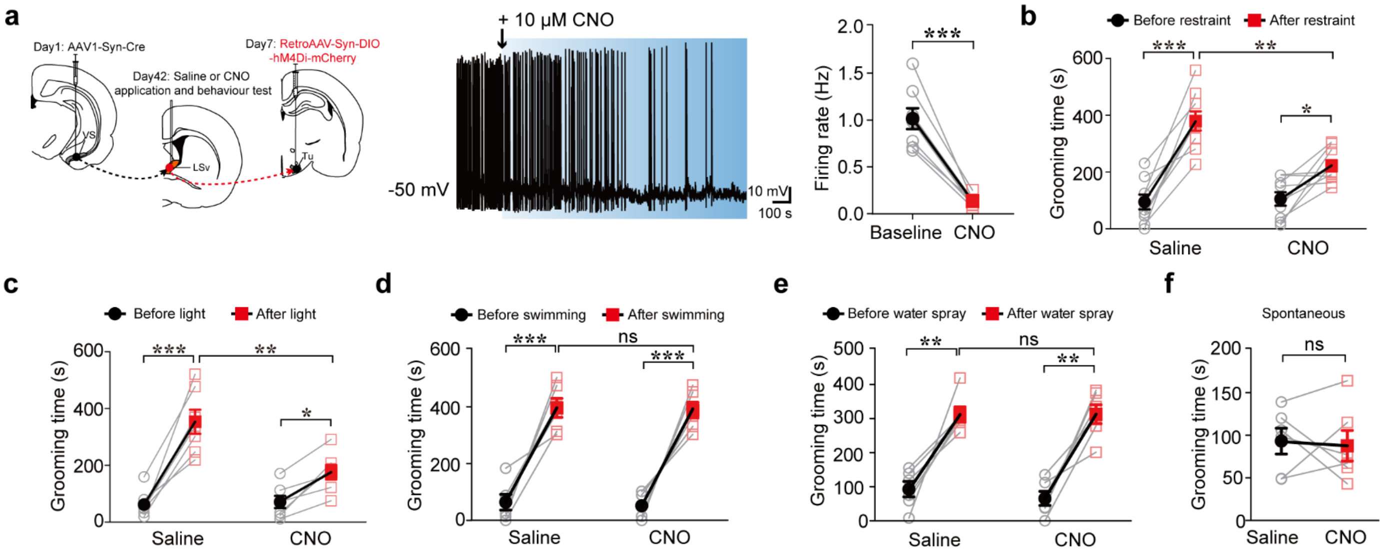

Supplementary Figure 6 | Chemogenetic inhibition of the VS→LSv→Tu circuit suppressed grooming caused specifically by emotional stress.

a, Left panel: Schematics for chemogenetics manipulation. Targeted functional inhibition of the VS→LSv→Tu was achieved by injection of trans-synaptic AAV1-Cre into VS, retroAAV-DIO-hM4Di-mCherry into Tu and delivery of CNO directly into LSv. Middle and right panel: Efficacy of inhibition was demonstrated in current-clamp recording of LSv in brain slices and superfusion of 10 μM CNO (blue shade); n=8 neurons from 3 rats; ****P* < 0.0001, Students paired two-tailed t-test. **b-e,** While significant increases in grooming time were found in saline-injected control group following restraint **(b,** Interaction ; F(1,8) = 9.166 , *P* = 0.0164 ; Optostimulation main effect ; F(1,8) = 16.14, *P* = 0.0039 ; Restraint model main effect ; F(1,8) = 73.52, *P* < 0.0001; Saline, ****P* = 0.0002; CNO, **P* = 0.0354; After restraint, ***P* = 0.0078 **)** and bright light exposure **(c,** Interaction ; F(1,6) = 23.6 , *P* = 0.0028 ; Optostimulation main effect ; F(1,6) = 10.45, *P* = 0.0178 ; Light model main effect ; F(1,6) = 57.75, *P* = 0.0003; Saline, ****P* < 0.0001; CNO, **P* = 0.0164; After restraint, ***P* = 0.0012**)** after CNO injection, the increases in grooming time were significantly smaller. In contrast, the increases in grooming time after swimming **(d,** Interaction ; F(1,5) = 0.006526 , *P* = 0.9387 ; Optostimulation main effect ; F(1,5) = 0.3062, *P* = 0.6039 ; Swimming model main effect ; F(1,5) = 69.63, *P* = 0.0004 ; Saline, ****P* = 0.0008; CNO, ****P* = 0.0007**)**, and water spray **(e,** Interaction ; F(1,5) = 0.3276 , *P* = 0.5981 ; Optostimulation main effect ; F(1,5) = 1.713, *P* = 0.2476 ; Water spray model main effect ; F(1,5) = 48.42, *P* = 0.0009; Saline, ***P* = 0.0028; CNO, ***P* = 0.0016**)**, were similar in both saline and CNO-injected groups. ns: non-significant; Two-way repeated measures ANOVA with Sidak *post-hoc* test**. (f)** CNO injection did not increase the time of spontaneous grooming. n = 6, *P* = 0.8020. ns: non-significant; Student's paired two-tailed t-test. All data are presented as mean ± SEM. Source data are provided as a 'Source Data File'.

Supplementary Table 1 (Related to Figure 1)

Figure	Parameter	Number	Statistical test	Significance level
1a	Grooming time	Control(n=4); Restraint(n=4)	Student's unpaired two-tailed t-test.	P = 0.0024
1b	Normalized c-Fos fluorescence intensity	Control(n=4); Restraint(n=4)	Student's unpaired two-tailed t-test	P = 0.0008 for LSv;<br>Not significant for LSd;
1d	Grooming time	8	One-way repeated measures ANOVA with Tukey *post-hoc* test	F (1.034,7.237) = 36.46, P = 0.0004;<br><br>Pre-Off vs. On, P = 0.0019;<br>On vs. Post-Off, P = 0.0008
1e	Delay time	8	Student's paired two-tailed t-test	P < 0.0001
1e	Time Spent	8	Student's paired two-tailed t-test	P = 0.0041
1f	Grooming time	self-grooming (n=3)	One-way repeated measures ANOVA with Tukey *post-hoc* test	F (1.322,2.643) = 587.3, P = 0.0003
1f	Grooming time	social grooming (n=3)	One-way repeated measures ANOVA with Tukey *post-hoc* test	F (1.000, 2.000) = 1.000, P = 0.4226;
1f	Grooming time	self-grooming (n=3); social grooming (n=3)	Student's paired two-tailed t-test	self-grooming *vs* social grooming during light on period, P < 0.0001
1g	Grooming time	LSv group (n=4)	One-way repeated measures ANOVA with Tukey *post-hoc* test	F (1.09, 3.271) = 26.76, P =0.110
1g	Grooming time	LSd group (n=4)	One-way repeated measures ANOVA with Tukey *post-hoc* test	F(1.628,4.883)= 0.1304, P = 0.8427
1g	Grooming time	LSv group (n=4); LSd group (n=4)	Student's paired two-tailed t-test	LSv group and LSd group comparison during light on period, P < 0.0001
1h	Grooming time	LSv unilateral group (n=5)	One-way repeated measures ANOVA with Tukey *post-hoc* test,	F (1.022, 4.089) =154.6, P = 0.0002
1h	Grooming time	LSv bilateral group (n=4)	One-way repeated measures ANOVA with Tukey *post-hoc* test	F (1.026, 3.079) = 326.8, P = 0.0003
1h	Grooming time	LSv unilateral group (n=5); LSv bilateral group (n=4)	Student's two-tailed t-test	For LSv unilateral group and LSv bilateral group comparison during light on period, P = 0.0553

Supplementary Table 2 (Related to Figure 5)

Figure	Parameter	Number	Statistical test	Significance level
5b	Grooming time	n=9 for optogenetic (OS) group; n=10 for body restraint (RS) group; n=8 for light exposure (LS) group; n=8 for swimming (SM) group; n=9 for water spray (WS) group; n=14 for spontaneous (ST) group	One-way ANOVA with Tukey *post-hoc* test	$F (5, 52) = 54.32$, $P < 0.0001$;<br><br>ns: not significant;<br>OS vs. ST, $P < 0.0001$;<br>OS vs. WS, $P = 0.0387$;<br>OS vs. SM, $P < 0.0001$
5c	Grooming bouts	n=14 rats for spontaneous group, n=9 rats for water spray group, n=8 rats for swimming group, n=8 rats for light exposure group, n=10 rats for body restraint group, n= 9 rats for optogenetic group	One-way ANOVA with Tukey *post-hoc* test	$F (5, 60) = 19.85$, $P < 0.0001$;<br><br>ST vs. RS, $P < 0.0001$;<br>ST vs. LS, $P < 0.0001$;<br>ST vs. WS, $P < 0.0001$;<br>ST vs. OS, $P < 0.0001$;<br>RS vs. SM, $P = 0.0182$;<br>LS vs. SM, $P = 0.0160$;<br>WS vs. SM, $P = 0.0125$;<br>SM vs. OS, $P = 0.0045$
5d	Single bout duration	n=14 rats for spontaneous group, n = 9 rats for water spray group, n=8 rats for swimming group, n = 8 rats for light exposure group, n=10 rats for body restraint group, n =9 rats for optogenetic group	One-way ANOVA with Tukey *post-hoc* test	$F (5, 52)= 27.93$, $P < 0.0001$;<br><br>ST vs. WS, $P = 0.0031$;<br>ST vs. SM, $P < 0.0001$;<br>RS vs. SM, $P < 0.0001$;<br>LS vs. SM, $P < 0.0001$;<br>WS vs. SM, $P < 0.0001$;<br>SM vs. OS, $P < 0.0001$
5e	Transitions per bout	n=14 rats for spontaneous group, n= 9 rats for water spray group, n=8 rats for swimming group, n=8 rats for light exposure group, n=10 rats for body restraint group, n =9 rats for optogenetic group	One-way ANOVA with Tukey *post-hoc* test	$F (5, 39) = 6.174$, $P = 0.0003$;<br><br>ST vs. RS, $P = 0.0093$;<br>ST vs. LS, $P = 0.0029$;<br>ST vs. OS, $P = 0.0054$

Supplementary Table 3 (Related to Figure 9)

**Figure**	**Parameter**	**Number**	**Statistical test**	**Significance level**
9c	Grooming time	n = 9	Two-way repeat measures ANOVA with Sidak *post-hoc* test	F(1,8) = 34.73 , *P* = 0.0004; Optostimulation main effect; F(1,8) = 5.46, *P* = 0.0477; Restraint model main effect; F(1,8) = 47.42, *P* = 0.0001; Off, *P* < 0.0001; On, P=0.0026; After restraint, *P* = 0.0001
9d	Grooming time	n = 9	Two-way repeat measures ANOVA with Sidak *post-hoc* test	F(1,8) = 5.475, *P* = 0.0474; Optostimulation main effect; F(1,8) = 9.522, *P* = 0.015; Light model main effect; F(1,8) = 75.05, *P* < 0.0001; Off, *P* < 0.0001; On, *P*=0.0034; After restraint, *P* = 0.0166
9e	Grooming time	n = 6	Two-way repeat measures ANOVA with Sidak *post-hoc* test	F(1,5)=0.05888, *P* = 0.8179; Optostimulation main effect; F(1,5) = 0.05885, *P* = 0.818 ; Swimming model main effect; F(1,5) = 42.09, *P* = 0.0013; Off, *P* = 0.0052; On, *P* =0.0040
9f	Grooming time	n = 6	Two-way repeat measures ANOVA with Sidak *post-hoc* test	F(1,5)= 0.1959 , *P* = 0.6766; Optostimulation model main effect; F(1,5)=0.1837,*P*= 0.6861; Water spray main effect; F(1,5) = 124.8, *P* = 0.0001; Off, *P*=0.0033; On, *P* =0.0022
9g	Grooming time	n=6	Student's paired two-tailed t-test	*P* = 0.4861
9h	Firing Frequency	n=11	One-way repeated measures ANOVA with Tukey *post-hoc* test	F(1.529, 15.29)=19.60, *P*=0.0001; Control vs. CNO, *P* = 0.0021; CNO vs. CNO+Yellow light, *P* = 0.0021

Supplementary Table 4 The oligonucleotide primers used for single-cell RT-PCR:

Gene	Sequence	Position	Product length (bp)	Accession No.
vGluT2	se: GAG CCC CGC AAA GCA TC	798	425	AF271235
	as: CTC GGG GCA ATA TCC AAG TG	1300		
	nese: TCA AGA CCC CAT GGA GGA AGT	896	283	
	neas: GTT CAT GAT CTT TCG CAC TGT AG	1145		
GAD67	se: ACC CTG GTG CCC GCT TCC	244	390	M76177
	as: TAT TGG TAT TGG CAG TTG ATG TC	611		
	nese: GGA CTT CCA CCA CCC ACA C	413	185	
	neas: CTA AAC CAA TGA TAT CCA AAC CAG	574		

Oligonucleotide primers used for single-cell RT-PCR

Position 1 is the first nucleotide of the initiation codon. ‘se’ and ‘as’ mark sense and antisense primers. ‘nese’ and ‘neas’ correspond to sense and antisense nested primers, accordingly.
