# Supplementary material for: A limbic circuitry involved in emotional stress-induced grooming
Source: Nat Commun. 2020 May 8;11:2261. doi: 10.1038/s41467-020-16203-x (PMC7210270; doi:10.1038/s41467-020-16203-x)
Supplement: Supplementary file 2 — Description of Additional Supplementary Files [file 41467_2020_16203_MOESM2_ESM.pdf]

Description of Additional Supplementary Files

File: Supplementary Movie 1

Description: Example showing that optogenetic stimulation of LSv but not LSd in the same animal triggers self-grooming, typically preceded by rearing-like arousal behavior.

File: Supplementary Movie 2

Description: Example showing that optogenetic stimulation of LSv leads to self-grooming but not social grooming.

File: Supplementary Movie 3

Description: Optogenetic activation of the precise VS→LSv→Tu circuitry results in robust self-grooming free of rearing like arousal behavior
