# Supplementary material for: A limbic circuitry involved in emotional stress-induced grooming
Source: Nat Commun. 2020 May 8;11:2261. doi: 10.1038/s41467-020-16203-x (PMC7210270; doi:10.1038/s41467-020-16203-x)
Supplement: Supplementary file 6 — Reporting Summary [file 41467_2020_16203_MOESM6_ESM.pdf]

nature research

Corresponding author(s): Y. Ke

Last updated by author(s): Apr 6, 2020

Reporting Summary

Nature Research wishes to improve the reproducibility of the work that we publish. This form provides structure for consistency and transparency in reporting. For further information on Nature Research policies, see Authors & Referees and the Editorial Policy Checklist.

Statistics

For all statistical analyses, confirm that the following items are present in the figure legend, table legend, main text, or Methods section.

n/a	Confirmed	
☐	☒	The exact sample size (*n*) for each experimental group/condition, given as a discrete number and unit of measurement
☐	☒	A statement on whether measurements were taken from distinct samples or whether the same sample was measured repeatedly
☐	☒	The statistical test(s) used AND whether they are one- or two-sided<br>*Only common tests should be described solely by name; describe more complex techniques in the Methods section.*
☒	☐	A description of all covariates tested
☒	☐	A description of any assumptions or corrections, such as tests of normality and adjustment for multiple comparisons
☐	☒	A full description of the statistical parameters including central tendency (e.g. means) or other basic estimates (e.g. regression coefficient) AND variation (e.g. standard deviation) or associated estimates of uncertainty (e.g. confidence intervals)
☐	☒	For null hypothesis testing, the test statistic (e.g. *F*, *t*, *r*) with confidence intervals, effect sizes, degrees of freedom and *P* value noted<br>*Give P values as exact values whenever suitable.*
☒	☐	For Bayesian analysis, information on the choice of priors and Markov chain Monte Carlo settings
☒	☐	For hierarchical and complex designs, identification of the appropriate level for tests and full reporting of outcomes
☐	☒	Estimates of effect sizes (e.g. Cohen's *d*, Pearson's *r*), indicating how they were calculated

Our web collection on statistics for biologists contains articles on many of the points above.

Software and code

Policy information about availability of computer code

Data collection	For electrophysiology experiments, data acquisition was performed off-line using custom Clampfit software (version 10.7.0.3). For behavioral experiments, the behaviours of the animals were recorded by one or more cameras (C922 PRO STREAM WEBCAM,Logitech, Hong Kong), the position of the animal was monitored using a commercially available video tracking software (ANY-Maze,version 4.7, Stoelting CO, USA). MATLAB (Mathworks, Natick, MA, Version R2017a) were used in the photometry recording experiments and behavioral analysis. Anatomy data were acquired using a commercial confocal microscope (Nikon C1), operated with Image J Software (v.1.46).
Data analysis	GraphPad Prism 7.02, Clampfit 10.7, Photoshop CC 2017, Adobe Illustrator CS6 (version 16), Microsoft Excel 365 and custom MATLAB codes (Mathworks, Natick, MA, Version R2017a) were used to analyze data. Custom codes for fibre photometry data analysis are available from the corresponding author upon request.

For manuscripts utilizing custom algorithms or software that are central to the research but not yet described in published literature, software must be made available to editors/reviewers. We strongly encourage code deposition in a community repository (e.g. GitHub). See the Nature Research guidelines for submitting code & software for further information.

Data

Policy information about availability of data

All manuscripts must include a data availability statement. This statement should provide the following information, where applicable:

- Accession codes, unique identifiers, or web links for publicly available datasets
- A list of figures that have associated raw data
- A description of any restrictions on data availability

Raw data and codes that support the findings of this study are available from the corresponding author upon reasonable request.

Field-specific reporting

Please select the one below that is the best fit for your research. If you are not sure, read the appropriate sections before making your selection.

☒ Life sciences ☐ Behavioural & social sciences ☐ Ecological, evolutionary & environmental sciences

For a reference copy of the document with all sections, see nature.com/documents/nr-reporting-summary-flat.pdf

Life sciences study design

All studies must disclose on these points even when the disclosure is negative.

Sample size	No statistical methods were used to calculate the sample size. Sample size was determined based on our experience and the published studies of the similar field. PMID: 29302029; PMID: 31371721; PMID: 27306314.
Data exclusions	Animals in which post-hoc histological examination showed that viral targeting or the position of implanted fiber / cannulas were in the incorrect location were excluded from analysis.
Replication	All key results were replicated in multiple animals, and the attempts at replication were successful based on at least three independent experiments.
Randomization	Animals were randomly assigned to experimental group.
Blinding	For the surgeries and the electrophysiological recordings, they were performed by the same experimenters so they were not blind to the experiments. For optogenetic and pharmacological manipulations, an experimenter was blind to the treatment condition during behavioral scoring, which was automated to ensure unbiased data analysis. Group allocations were randomized during data collection.

Reporting for specific materials, systems and methods

We require information from authors about some types of materials, experimental systems and methods used in many studies. Here, indicate whether each material, system or method listed is relevant to your study. If you are not sure if a list item applies to your research, read the appropriate section before selecting a response.

Materials & experimental systems

n/a	Involved in the study	
☐	☒	Antibodies
☒	☐	Eukaryotic cell lines
☒	☐	Palaeontology
☐	☒	Animals and other organisms
☒	☐	Human research participants
☒	☐	Clinical data

Methods

n/a	Involved in the study	
☒	☐	ChIP-seq
☒	☐	Flow cytometry
☒	☐	MRI-based neuroimaging

Antibodies

Antibodies used	Rabbit monoclonal anti-Fos antibody (Cell Signaling Technology, #2250) was used at 1:1000, Alexa Fluor 488 goat anti-rabbit (Invitrogen, A-11034) was used at 1:1000.
Validation	All antibodies were purchased from vendors in the USA. These products are normally quality controlled at the company. Furthermore, the antibodies were validated and used in previous studies.<br>1. de Guglielmo, G. et al. Inactivation of a CRF-dependent amygdalofugal pathway reverses addiction-like behaviors in alcohol-dependent rats. Nature communications 10, 1238, doi:10.1038/s41467-019-09183-0 (2019).<br>2 Cassidy, R. M. et al. A lateral hypothalamus to basal forebrain neurocircuit promotes feeding by suppressing responses to anxiogenic environmental cues. Science advances 5, eaav1640, doi:10.1126/sciadv.aav1640 (2019).

Animals and other organisms

Policy information about studies involving animals; ARRIVE guidelines recommended for reporting animal research

Laboratory animals	Adult male Sprague-Dawley (SD) rats were used for all experiments. When performing viral injections, we generally used 300-320g animals (8-10 week old rats).
Wild animals	This study did not involve wild animals.

Field-collected samples	This study did not involve samples collected from the field.
Ethics oversight	All animals were handled in strict accordance with The Chinese University of Hong Kong (CUHK) guidelines and the procedures were approved by the Animal Experimentations and Ethics Committee.

Note that full information on the approval of the study protocol must also be provided in the manuscript.
